# The evolution of phonemic verbal fluency test: bridging tradition with contemporary insights

**DOI:** 10.3389/fpsyg.2026.1774256

**Published:** 2026-07-17

**Authors:** Olga Morkovina, Anastasia Ziberova, Olga Sapunova, Piruza Manukyan, Anastasia Sharapkova

**Affiliations:** 1 Department of English, Faculty of Computational Mathematics and Cybernetics, Lomonosov Moscow State University, Moscow, Russia; 2 Department of General Psychology, Faculty of Psychology, Lomonosov Moscow State University, Moscow, Russia; 3 Department of Verbal Arts, Faculty of Arts, Lomonosov Moscow State University, Moscow, Russia; 4 Laboratory of Diagnostics and Advancing Cognitive Functions, Research Institute for Brain Development and Peak Performance, RUDN University, Moscow, Russia; 5 Department of English Linguistics, Faculty of Philology, Lomonosov Moscow State University, Moscow, Russia

**Keywords:** executive function, lexical access, mental recall, neuropsychological tests, phonemic verbal fluency, prefrontal cortex, speech production, verbal behavior

## Abstract

Phonemic verbal fluency tests are a long-standing part of neuropsychological assessment. Although these tests are widely used to assess an individual's executive control and lexical retrieval potential, theoretical complexity behind them has not been sufficiently addressed in literature. Total word count remains the common metric, as earlier theoretical assumptions had shaped how the paradigm was operationalized. However, research and clinical practice have gradually revealed the insufficiency of this metric. This review aims to disentangle the test's interdisciplinary underpinnings by analyzing its psychological, linguistic, and neuroscientific foundations. Our assessment of the verbal fluency test's application uncovers apparent discrepancies between the conventional approach to interpreting test's data and recent paradigmatic shifts within each discipline. Having identified the critical discrepancy between the conventional interpretation of verbal fluency data and contemporary paradigmatic shifts across these disciplines we argue that phonemic fluency assessment should move beyond the total score to a systematic analysis of rich and variable response patterns, which may offer valuable insights into complex cognitive-linguistic processes. To conclude, embracing the inconsistencies between the current understanding of the cognitive processes measured by the test and its historical foundations may increase its diagnostic and research value.

## 1 Introduction

Verbal fluency (VF) tests have long been an integral part of neuropsychological assessment in clinical and research settings worldwide. Its most common types—frequently but not necessarily administered together—are semantic and phonemic fluency tests. While the former requires participants to name items from a given category such as animals, the latter involves listing words that start with a particular letter (typically FAS, CFL, or PRW). The total number of produced words remains the most common measure of test performance; however, new parameters such as sum scores, error types, speech breaks, and semantic relatedness have emerged recently (Amunts et al., 2021). Since the two fluency paradigms supposedly rely on different linguistic, neuropsychological, and neural underpinnings (Biesbroek et al., 2016; Glikmann-Johnston et al., 2015; Olmos-Villaseñor et al., 2023; Shao et al., 2014) and have evolved largely independently, in this review we trace the development of phonemic fluency (PVF) at the background of its contemporary theoretical frameworks.

Remarkable time efficiency and ease of administration have made VF tests widely popular for research and diagnostic purposes. VF tests have been applied to provide information on verbal abilities, including lexical access (Carr et al., 2018) and vocabulary size (Dubé, 2021), semantic and lexical memory structure (Moraes et al., 2013) and architecture of semantic networks (Wille, 2020). It has long been used to measure executive functions (EF) and is even referred to as an “executive” language task (Aita et al., 2019; Cipolotti et al., 2021), as it requires the inhibition of irrelevant words (Henry et al., 2015), efficient use of working memory (Beausoleil et al., 2003), cognitive flexibility (Rende et al., 2002), and switching ability (Abwender et al., 2001). Considered particularly challenging from the cognitive standpoint (Chouiter et al., 2016), VF tests have been studied in patients with various types of cognitive impairment, including Alzheimer's disease (Henry et al., 2004; Monsch et al., 1992), Mild Cognitive Impairment (Nutter-Upham et al., 2008), Parkinson's disease (Henry and Crawford, 2004a), Traumatic Brain Injuries (Henry and Crawford, 2004b), schizophrenia (Frith et al., 1995; Riva et al., 2000), as well as in specific non-clinical populations such as children (Regard et al., 1982; Riva et al., 2000), adults of an advanced age (González-Burgos et al., 2019) and bilinguals (Grogan et al., 2009; Patra et al., 2020), bringing under the microscope various demographic parameters of the cohorts, such as age (Elgamal et al., 2011; Rodríguez-Aranda and Martinussen, 2006), sex (Hirnstein et al., 2023), and the level of education (Ilkmen and Soncu Büyükişcan, 2022; Tombaugh et al., 1999). Such extensive research most naturally resulted in the tests' spreading beyond clinical practice.

Since VF tests have been a constituent part of clinical testing practice for over a century, at some point the testing procedure crystallized and became immune to paradigm controversies. However, profound advances in psychology, linguistics and neuroscience are calling for a more informed approach toward interpreting VF test results. Inevitably, it advanced further the test procedure, resulting in a range of review and meta-analytical publications on PVF in different languages (Villalobos et al., 2023), demographic groups (Arán Filippetti et al., 2023; Cermak et al., 2021; Hirnstein et al., 2023; Wajman, 2020), conditions (Bauer and Malek-Ahmadi, 2023; Grissom et al., 2024; Olmos-Villaseñor et al., 2023; Raucher-Chéné et al., 2017; Wright et al., 2023; Yeung and Lin, 2021), specific abilities (Giovannoli et al., 2023) and research paradigms (Ren et al., 2024; Tassi et al., 2022). Apart from the studies focusing on the cognitive and linguistic mechanisms possibly underlying fluency (e.g., Luo et al., 2010; Shao et al., 2014; Whiteside et al., 2016), recent papers highlight physiological aspects of VF performance (Challakere Ramaswamy and Schofield, 2022; Gullsvåg and Rodríguez-Aranda, 2023). Historical and methodological roots of fluency research have also earned some attention when the intrinsically embedded discrepancies in the underlying theoretical constructs started to pop up in practice (e.g., De Oliveira Santana and Dos Santos, 2015; Ruff et al., 1996).

The growing data collected worldwide still requires a bird's eye view of the test's conceptual history. Although the analytical methods have grown more sophisticated, the conceptual pillars of the test remain embedded in all modern re-interpretations. This narrative review aims to trace the evolution of theoretical concepts underlying VF results' interpretation, show the potential discrepancies arising from specific theoretical and/or methodological viewpoints, and suggest possible vistas for future reinterpretation. Striving to cover a wide range of studies in the field and detail all the theoretical constructs, we conducted the initial search across PubMed and Google Scholar, using combinations of the keywords, allowing for possible variations (e.g., “phonemic fluency”, “verbal fluency”, and “phonemic verbal fluency”). Additionally, the most prominent articles listed in relevant reference works were included in the analysis. Finally, we used search tools such as Litmaps, Connected Papers, Semantic Scholar and ResearchRabbit to broaden the scope and establish possible links between papers. For the supplementary tables, a more rigorous yet less wide-reaching method was employed (to be discussed below).

To the best of our knowledge, this is the first attempt at integrating several perspectives to evaluate the diagnostic and research potential of the PVF test. First, we trace the test's history to outline the theoretical assumptions that underlie its current shape, including psychometrics, neuropsychological research into executive functions, neuroscience, psycho- and neurolinguistics. Next, these assumptions are placed within the context of their respective disciplines, and the evolution of each is described to evaluate their current relevance. Finally, the paradigm is viewed in the context of state-of-the-art research, and implications for its further use are considered. Several contemporary approaches are briefly outlined, with a focus on the most conventional fluency metric, which has remained in use throughout the history of the test: the total score. Those more recent—and seeming more promising—we will address in further publications, while this study we provide bottom-up and top-down approaches to provide a comprehensive analysis, encompassing both theoretical and methodological levels.

## 2 Verbal fluency testing within the context of its time: fluency theories shaken not stirred

**Figure 1:**
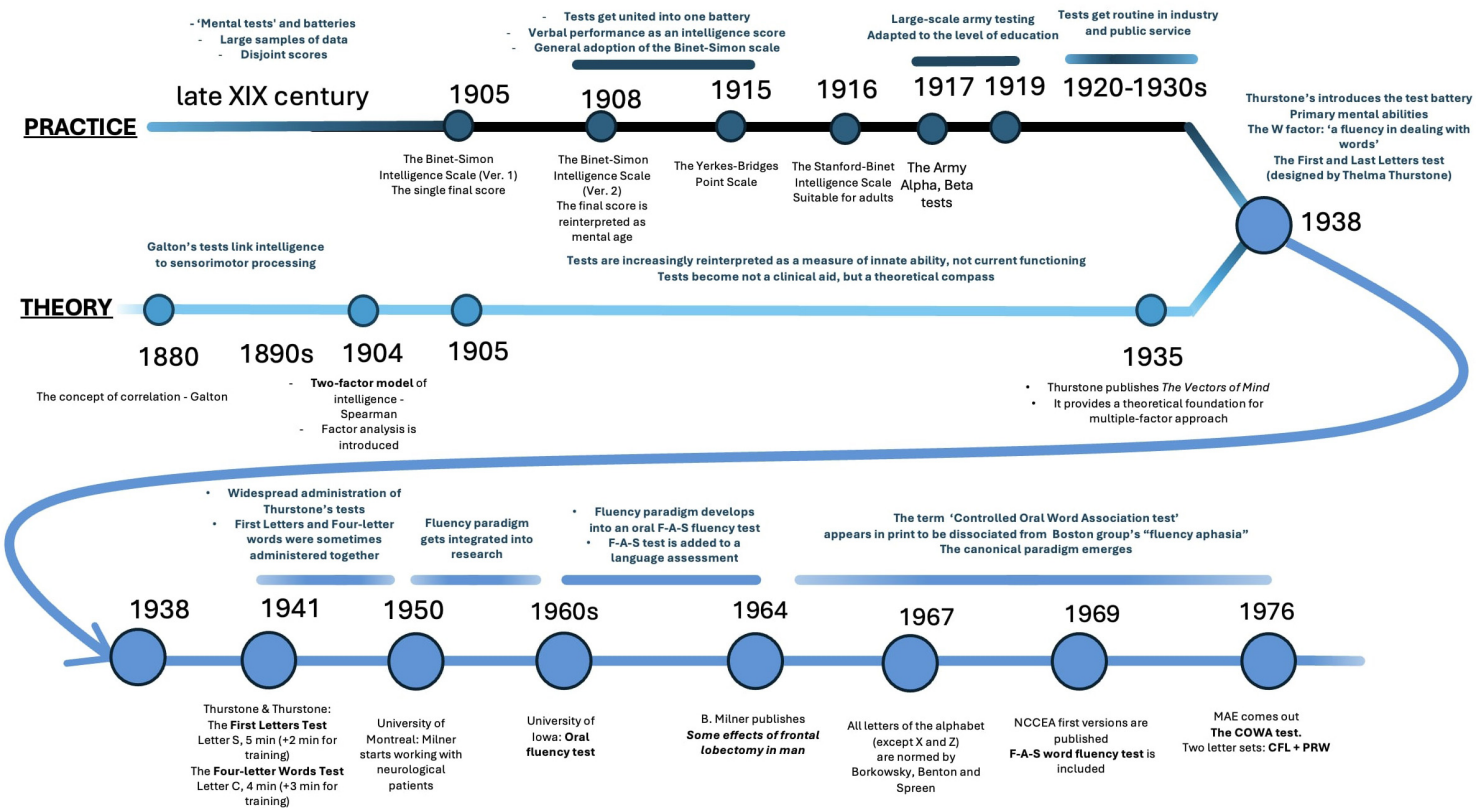
Theoretical and methodological evolution of the phonemic verbal fluency paradigm. Horizontal lines above the timeline indicate time periods. Dots along the line indicate the milestones.

Current PVF tests are commonly traced back to A. Benton's works (e.g., Benton, 1968, 1967) or even earlier—L.L. Thurstone's batteries (Primary Mental Abilities: Thurstone, 1938; Thurstone and Thurstone, 1941). Yet the concept of “verbal fluency” emerged from a broader and more diverse context of the late 19th—early 20th century theories. Its journey from a component of early intelligence theory to a standardized clinical tool has been shaped by both practical necessity and lasting ambiguities surrounding the very term “fluency”. The major milestones are visualized in Figure 1.

Following several attempts at proper operationalization of intelligence (Cattell, 1890, in: Cattell, 2025; Cattell and Farrand, 1896; Galton, 1885, 1888; Jastrow, 1893; Pearson, 1904; Spearman, 1904), conceptualizing it as a sensorimotor capacity, proved to be oversimplistic (Wissler, 1901). Sensorimotor tests ultimately failed to correlate with academic achievement rendering them unsuitable for informing the compulsory education system (Aiken, 1996; Wasserman and Kaufman, 2016; Ackerman, 2022). The possible conceptual link between mental ability and language as proposed by (Esquirol 1838) was not yet implemented in practice. Thus, to provide objective measures of intellectual performance, the first widely used intelligence measurement tool was designed: the Binet-Simon scale (1905, 1908, 1911; Gould, 1981). This scale was an extensive series of verbal and non-verbal tasks that mirrored other batteries (e.g., Ebbinghaus, 1897; Rieger, 1889), yet it marked a critical change by grounding the measurement of intelligence in verbal performance. Unlike Galton's and Cattell's mental tests, the battery relied heavily on verbal performance. This included semantic (giving definitions, naming objects) and syntactic competence (building a sentence with the given words), as well as a prototype of what is currently known as “unconstrained verbal fluency”, or “free fluency”: listing random words for 3 min (Aiken, 1996; Martz et al., 2022; Weiner et al., 2021). The latter paradigm might have been indirectly related to the free association method (pioneered by Freud and modified by Jung).

The concept of intelligence behind these tasks evaded strict definition, since the battery was designed to supplement judgment of a pedagogical trajectory rather than explore the concept *per se*. Yet it was quickly reinterpreted as the one measuring innate capacity, and since the diversity of data obtained by large-scale testing suggested more fine-grained cognitive mechanisms, theories of separate mental abilities began to arise. Soon the test was translated into English and adapted for use in America, where it grew especially popular (Goddard, 1910, 1908; Yerkes, 1915) to be then revised into Stanford-Binet battery (Terman, 1916). Numerous ability tests of the 1920s and 1930s were modeled after the Binet-Simon or the Stanford-Binet scale. Adoption of a point scale instead of an age scale allowed performance to be measured numerically, paving the way for more differentiated functional assessment.

Fast forward to the 20th century, when intelligence theories grew more factorized. The first milestone was a two-factor theory, which explained test performance *via* the “general intelligence” and more “specific” task-related factors (Spearman, 1927, 1904). L.L. Thurstone suggested another model of abilities that represented independent group factors, revealed through a series of intercorrelated sub-tests (Thurstone, 1931, 1934, 1935, 1938). His own experience as a contributor to the first version of the 1917 Army intelligence test (Yoakum and Yerkes, 1920) and, later, a college entrance test (Thurstone, 1919), allowed him to develop a formally similar series of assessments within a different—analytical—framework, which separated form from content. Within it, he postulated a specific ability: “fluency in dealing with words… separate from… ideas and meanings”, distinct from nonverbal fluency (Thurstone, 1938) or, as it was rephrased later,

“the ability to produce words irrespective of their semantic content.” (Fruchter, 1948)

Counterintuitive as it may seem from the current point of view, a structuralist trend toward dealing with words separately from their meaning and context mirrored a similar one in linguistics: the structure of language was viewed separately from the structure of thought (Bloomfield, 1933). This was a viable attempt to formalize the approach to language activity and make it experimentally measurable.

The tests that measured “word fluency” (the W-factor) mostly focused on orthographic knowledge of separate words. One of them was the First Letters test, which required participants to write words starting with a given letter. It was published as one of the Chicago tests of primary mental abilities (Thurstone and Thurstone, 1941), adapted for various ages (the SRA battery for children: Thurstone and Thurstone, 1947) and used to test learners' and candidates' aptitudes and personality traits (Adjutant General's Office, U.S. Army, 1954; Carter and Nixon, 1949; Gibb, 1947; King, 1957; Martin, 1958; Swineford, 1949). Apparently, by the 1950s its use had spread into novel domains: personality research beyond educational or employment purposes (Blewett, 1954; Clements, 1954) and, most importantly, clinical studies (Milner, 1964; Wilkins, 1959).

The written form was soon found challenging for patients with brain lesions, which were often elderly, less educated and affected by additional physical conditions such as writing hand paresis (Ruff et al., 1996). As a result, the oral controlled verbal fluency task (which would soon become known as COWAT) was developed at the University of Iowa (Bechtoldt et al., 1962; Fogel, 1962, 1960): first using the letters F-A-S (the choice lacking an explicit justification in print), then standardizing two sets of mixed “easy” and “difficult” letters based on language frequency and productivity: C-F-L and P-R-W (Benton and Hamsher, 1976; Spreen and Benton, 1969). Since then, the fluency test has become an integral part of various clinical batteries (Figure 1).

In fact, the term “fluency” itself is ambiguous as its colloquial meaning related to speech flow existed long before and parallel to its terminological definition. Sir Francis Galton was among the first to apply it to mental functioning (Rogers, 1953), linking it to quick association-based retrieval of ideas (Galton, 1883). The apparently non-terminological use of fluencyrelied on the intuitively grasped dictionary meaning—which was, however, mostly speech-related (e.g., Webster, 1898: “Fluent: ready with words; voluble; uttering with facility; hence, of language, flowing; fluency: quality of being fluent”; Murray, 1913: “Fluent: of speech, style, etc.: Flowing easily and readily from the tongue or pen; Fluency: a smooth and easy flow; readiness, smoothness; esp. with regard to speech”). Later, the Boston aphasia classification retained it (“fluency in conversation”: e.g., Benson, 1967; Buckingham and Kertesz, 1974; Goodglass et al., 1964, 1966, 1967; see also the Western Aphasia battery: Kertesz, 1982, 1979). A similar use of “fluency” has long been common to second language acquisition, language pedagogy and developmental studies of reading and writing (Branscom, 1942; Burt, 1917; López-Escribano et al., 2022; Peltonen et al., 2025; Pham, 2021; Voelker, 1944).

Even as Thurstone sought to narrow it down, the concept of fluency remained contested with research in other fields, where related constructs emerged. In personality research, fluency retained a broader cognitive meaning: “fluency of association”, reflecting such temperamental traits as “quickness of thinking, width and range in association and imagination” (e.g., Cattell, 1936), or “a measure of the rapidity of associative processes” (Notcutt, 1943). Soon the concept explicitly opposed to Thurstonian word fluency was added: “ideational fluency”, a quantitative

“facility in expressing ideas by the use of words and their meanings.” (Taylor, 1947)

The possibility of a single fluency factor responsible for verbal and non-verbal performance, originally ruled out by Thurstone, was debated for a while—perhaps because of the ambiguity of contemporary test batteries, which upon closer inspection revealed more than one type of fluency (Fruchter, 1948; Taylor, 1947). Thus, the concept of “word fluency” has always been surrounded with several closely related ones, whether terminologically or theoretically.

The discrepancy between various approaches to fluency did not pass unnoticed, and stimulated further theoretical debates about its true nature. Once the Boston Group included the “fluent/nonfluent” dimension into its “aphasia classification,” Benton's Controlled Verbal Fluency Task was renamed as Controlled Oral Word Association Test [COWA(T)] to avoid confusion. Still, the generic dictionary-based understanding of fluency could unwittingly interfere. For instance, Thurstone's tests were sometimes used to assess

“at the verbal level, the free play of association assumed to be a part of creative thinking” (Springbett, 1957),

or “the ability to write and talk easily” (Becker, 1948)—clearly not implied by his original framework. Far-fetched as it may sound, the 21st-century attempts to contrast verbal fluency against discourse fluency measures (Lofgren and Hinzen, 2022; Raymer et al., 2001) might have also been spurred by the long-standing vagueness of the original term.

The early history of the PVF test reveals the features that would define its application and interpretation for decades. Passed down through generations of researchers, it has mostly lost its original connections: the debate about intelligence and personality, the close-knit family of other tests loading on the fluency factor. As a separate paradigm, it could be freely coupled with other tests, yet once decoupled from its methodological foundations, it could be susceptible to misinterpretation or methodological losses.

The simplicity of Thurstone's “word fluency” factor was both its strength and its weakness, inviting immediate skepticism about its rigor and driving its adoption in neuropsychology. Even Thurstone's contemporaries providentially suspected that other functions should have been involved in fluency performance (Fruchter, 1948). While it was partly due to terminological vagueness, the field was ripe for a broader search—soon started in clinical studies of brain damage. The practice of administering psychometric batteries in education or employment has been reconsidered since then (Arthur et al., 2001; Charles and Florah, 2021; Taylor and Radford, 1986); yet the tradition which gave birth to the fluency paradigm may have reinforced its status as a valuable clinical tool. As such, “word fluency” was taken on board by the nascent neuropsychology, where the previously disembodied concept got embodied in a brain localization.

## 3 The tangled roots of verbal fluency: from frontal lobe localization to executive and linguistic constructs

This section outlines the VFT's conceptually and methodologically twisted path from the early clinical studies to the common tripartite association: (pre)frontal cortex—executive functions—phonemic fluency.

The term “neuropsychology”, championed by Osler (1913), gradually came into use in the early 20-th century (e.g., Goldstein, 1939, 1934; Lashley, 1937). Earlier theories concerning the physiological basis of intelligence and behavior did not stand the test of time, still some evidence of behavioral disturbances in localized brain damage, including the frontal lobes, awaited interpretation (e.g., Carmichael et al., 1940). Reports covering the “American crowbar case” (the story of Phineas Gage: Ferrier, 1878; Harlow, 1868, 1848; Jacobsen, 1935; Judd, 1907), or the changes in behavior of tumor patients described in (Oppenheim 1891, 1890), were supplemented by the post-World War I and II clinical cases and surgery for tumors and epilepsy (including the now-infamous leucotomy: Krudop and Pijnenburg, 2015; Leblanc, 2019; Penfield, 1935). It was the latter that eventually linked the frontal cortex to a range of higher functions such as initiative and planning (Leblanc, 2019), even though earlier studies had underestimated its role in adults (Hebb, 1945; Hebb and Penfield, 1940; Milner, 2006). Ultimately, higher functions such as attention, planning, speech and behavior regulation, motivation, emotional regulation, initiative, flexibility, abstract reasoning, problem-solving, inhibition, and behavior control (Feuchtwanger, 1923; Goldstein, 1944; Luria, 1966a,b) were spatially redefined as “frontal” (see the terminological use Bianchi, 1895), following the conventions of anatomical terminology (e.g., “basal frontal syndrome” in ophthalmology: Abbott, 1931; Craig and Lillie, 1931; “frontal lobe syndrome”: Alford, 1933; Moersch, 1925). Meanwhile, the meaning of “frontal functions” demanded further conceptual refinement.

A formal definition was particularly sought for in psychology, where two key ideas evolved over time. First, the distinction between automatic and controlled processing was drawn [e.g., selective attention or “cognitive control” (Broadbent, 1958; Posner and Snyder, 1975; Shiffrin and Schneider, 1977)]. Then, most models postulated a central executive component, such as the “supervisory attentional system” (Norman and Shallice, 1986), “executive attention” (Posner, 1980) or the “central executive” component of the working memory (Baddeley, 1996; Baddeley and Hitch, 1974). These monolithic constructs, underspecified and rarely linked to neurological findings, were often labeled “executive”, following a non-terminological tradition, for example “the functioning of the executive organs of the ego”: Kardiner (1932); “the executive functions of the ego”—Kardiner (1936); “regions with different controlling or executive functions”—Adrian (1936); “executive functions (behavior)”—Kardiner (1945). The concept of “executive functions” as we know it did not yet exist, as the meaning of “executive” (similarly to “fluency”) was dictionary-based: (Webster 1898)—“qualifying for, concerned with, or pertaining to, the execution of the laws or the conduct of affairs”; Murray (1913)—“having the function of executing or carrying into practical effect”; compare: “actions are… characteristic performances which are mediated by the body [and] … belong to the executive functions” (Bentley, 1934); “moving and acting (the two great executive functions)” (Bentley, 1947). As a term, it apparently solidified in Lezak (1982)—perhaps under the conceptual (but not terminological) influence of Luria's translated works, which attributed the executive role to one of the “functional units” of the brain (Luria, 1980, 1973).

With respect to physiology, the term “executive” was buttressed by the prominent computer metaphor (which, in turn, had capitalized on the organizational meaning of the term “executive”: “primate frontal cortex—executive of the brain”: Pribram, 1973). Yet unlike “executive functions”, “frontal lobe functions” were conceptualized as multiple but interacting processes (Godefroy et al., 1999; Shallice, 1988). At the same 1962 symposium at the University of Pennsylvania, where B. Milner presented her report on frontal patients (published in Milner, 1964), Dr. H-L. Teuber discussed the complex functions of the frontal lobes, later described as “unity and diversity” (Garcia-Barrera, 2019; Teuber, 1972, 1964). This transferred from the neuroanatomical (“frontal”) level to the conceptual (“executive”) one, listing distinct functions such as inhibition, task switching, and working memory update with a shared general component, which later developed into a (“common EF factor”: Friedman and Miyake, 2017; Gustavson et al., 2019; Miyake and Friedman, 2012; Seer et al., 2021; Toh and Yang, 2022). Thus, the “unity and diversity” model reconciled the “unitary” psychological and “diverse” physiological perspectives.

Since both “frontal (lobe) functions” and “executive functions” implied higher-order cognitive control processes, the terms became interchangeable (e.g., Barek et al., 2022). The term “fronto-executive”, common to various fields, still preserves this fusion (“fronto-executive network”: e.g., Hellmuth et al., 2021; La Marra et al., 2022; “fronto-executive functions”: Neuwirth and Kolb, 2020; “fronto-executive dysfunction”: Jellinger, 2024). Though the conceptual discrepancy between the two terms has recently been pointed out (Burgess and Stuss, 2017; Miyake et al., 2000; Phillips, 1997), the practice persists, and conscious distinction between them remains rare (“frontal and executive impairment”: Henry et al., 2004). Consequently, the tasks associated with executive control, could as well alternatively be termed “executive” (Jurado and Rosselli, 2007; Moffitt and Henry, 1989; Neary et al., 1988; Phillips, 1997) or “frontal” (Grodzinsky and Diamond, 1992; Miller et al., 2013; Mole et al., 2023; Riva et al., 2005). Occasionally, the former is even considered a direct equivalent of the latter (e.g., Fisk and Sharp, 2004).

Phonemic fluency became the third link in the chain due to an increased clinical use of test batteries by the 1950s—early 1960s. While not every clinical study involved a Thurstonian fluency test, Thurstone himself discussed the connection between the W-factor and amnestic aphasia (Thurstone, 1940). Some of the earliest clinical fluency studies were carried out on brain-damaged patients with parietal/parieto-occipital (Bechtoldt et al., 1962; Fogel, 1962, 1960) and frontal lesions (Benton, 1968; Milner, 1964). Despite possible links between fluency performance and lesion types (Borkowski et al., 1967), the authors did not specifically associate PVF effects with focal damage, favoring more general cerebral effects (Fogel, 1962). In behavioral terms, fluency effects were ascribed to a “rather generalized higher-level language impairment” (Benton, 1968; Milner, 1964). However, the 1960s publications were later interpreted as decisive proof of “frontal functions” underlying “word fluency” (McFie, 1975; Perret, 1974). The terminological fusion of “executive” and “frontal”, inevitably included verbal fluency, as controlled access and strategic retrieval of vocabulary under experimental conditions has been viewed within the “automatic / controlled” dichotomy from the start (Borkowski et al., 1967). The classical tripartite association was finally forged: (pre)frontal cortex as the locus of executive functions assessed *via* PVF tests (not to mention a number of nonverbal tests).

Since then, none of the links in the chain has remained stable and well-defined—least of all, “executive functions”. More than 30 definitions have emerged over the last 50 years, and up to 68 specific “functions” are listed in literature, assessed with (possibly) more than 109 isolated tests—apart from comprehensive batteries (Baggetta and Alexander, 2016; Bunge, 2024; Goldstein and Naglieri, 2014; Packwood et al., 2011). In most studies, the executive demands of PVF are mapped onto the three “core” components of the “unity and diversity” model (Miyake et al., 2000). *Suppression/inhibition* (also known as *inhibitory control*, or *response inhibition*) is required to filter out irrelevant or previously given words. Moving between subcategories or word clusters united by some principle, which requires an active search for a test-relevant unit in semantic memory rather than passive recall, exemplifies *switching/shifting* between mental sets or tasks *[cognitive/mental flexibility, (mental) set shifting]*. Finally, checking for errors and rule adherence relies on keeping the instructions and previous responses in working memory and *updating* it (Miyake et al., 2000; Shao et al., 2014). This justifies the status of PVF test as an “executive task”, although the contribution of each function or correlation between fluency parameters and EF values remains largely unclear (e.g., Fitzpatrick et al., 2013).

Given this foundational ambiguity, choosing a theoretical framework remains a matter of convenience and convention. Despite its logical congruence and heuristic potential, the “unity and diversity” model originally emerged as a practical solution rather than an exhaustive theory (Duncan and Friedman, 2024). Alternative models vary in hierarchical complexity, use of behavioral or cognitive measures and affective and motivational components implicated (Baggetta and Alexander, 2016; Demetriou et al., 2019; Garcia-Barrera, 2019). There is no consensus on the number, internal structure, interconnection, and even validity of the existing “unity and diversity” functions (Karr et al., 2018; Löffler et al., 2024; Sambol et al., 2023). For instance, an extended “classical” model includes two hierarchical levels of executive functions without a central component (Diamond, 2013). Other components were—both “core” (Bunge, 2024; Starr et al., 2023) and “non-core” ones (reviewed in: Dias et al., 2024). Some of these studies feature PVF as a “conventional” executive aspect along with “unconventional” ones, such as fluid intelligence (Martin et al., 2021; Roca et al., 2014), self-reported insight (Ilkmen and Soncu Büyükişcan, 2024) or subjective mental effort (Lam and Marquardt, 2022). Hence, “fluency”, not necessarily divided into semantic and phonemic, may even be listed as an executive domain in its own right (Alvarez and Emory, 2006; Delis et al., 2001; Demetriou et al., 2019; Fisk and Sharp, 2004; Packwood et al., 2011; Snyder, 2013).

What is more, certain issues stayed beyond the scope of mainstream research when the tripartite link was forged. Since then they have come to the fore and started raising concerns: in particular, social and emotional functioning and, sometimes, cultural bias (e.g., Howieson, 2019; Suchy et al., 2017). Novel models encompass emotional as well as cognitive aspects—for example the “core strands”: (Kaufman, 2010); the “hot / cool” or “cold” executive functions' (Zelazo and Carlson, 2012; Zelazo and Müller, 2002); metacognitive/emotional (or motivational) executive functions (Ardila, 2008). The rise of big data has expanded the demographic knowledge base, boosting the study of development and aging. Resulting age-specific models may defy “unity and diversity”—for instance, in children, where “unity” ontologically precedes “diversity” (Brydges et al., 2012; Karr et al., 2018; Wiebe et al., 2011). Emergent social and affective neuroscience, in turn, underscored the role of testing conditions and social context: familiarity with test paradigms, cultural and language biases, mood variability, communication anxiety, and stress-induced arousal on timed tasks, even the supposedly non-emotional cognitive ones [Carvalho and Ready, 2010; Howieson, 2019; Kimura et al., 2020; (see Supplementary Table 1, Part 1)]. In clinical neuropsychology and neurorehabilitation, the holistic approach has capitalized on the patient's social adaptation rather than the deficits (Reddy, 2025). Similarly, theoretical focus has shifted to everyday functioning, promoting external and, more specifically, “ecological validity”. Traditional tests have never been checked for their ability to predict performance outside the lab its (Boyle et al., 2023; Pinto et al., 2025). Consequently, their construct and ecological validity have been questioned (Chan et al., 2008; Diaz-Orueta et al., 2020; Löffler et al., 2024).

The 21st-century measures explicitly aim at age awareness [the ROBBIA battery: Stuss and Alexander, 2007; the Preschool Executive Function Battery (PEFB): Garon et al., 2014] and a higher ecological validity [the Behavior Rating Inventory of Executive Function (BRIEF): Gioia et al., 2000 (school-age version); Roth et al., 2005 (adult version); Gioia et al., 1996 (preschool version); the Behavioral Assessment of the Dysexecutive Syndrome in Children (BADS-C): Emslie et al., 2003; the Behavior Assessment System for Children—Second Edition (BASC-2): Reynolds and Kamphaus, 2004]. However, their reliability remains questionable, and the quest for the “perfect test” continues (Henry and Bettenay, 2010; Robertson and Schmitter-Edgecombe, 2017; Suchy et al., 2022, 2024a,b). PVF tests are inherently problematic in this respect, as their strategies of word retrieval are counterintuitive by definition. Despite moderate ecological validity, their correlation with real-life behavior is dubious (Burgess et al., 2006; Crawford and Henry, 2005).

Since executive functions act as an intermediary between perception, attention (low-level functions) and cognition (higher-level ones: Starr et al., 2023), testing paradigms are prone to task impurity—and, simultaneously, trapped in a methodological “vicious circle”. On the one hand, most models result from testing data and its parameters: the number of the tests used, their range, reliability and validity (Denckla, 1996; Karr et al., 2018). On the other hand, some constructs that underlie the tests may have lost their validity over the years (Chan et al., 2008). Phonemic fluency in particular was theoretically explained only after the paradigm had already been developed, which is a questionable premise even compared to other executive tests.

To resolve these issues, a wider range of tests could be used to probe for each function. At least two are needed to establish correlations, with sufficient convergent validity and explicitly identical theoretical underpinnings (Chan et al., 2008; Paap et al., 2015). Given the long and convoluted history of most neuropsychological constructs, the latter is not always feasible (see the examples of studies and their evaluation in Supplementary Table 1, Part 1). Meanwhile, diversity of tasks may blur fine distinctions. The latent variable approach navigates the issues by distinguishing between general, domain-specific and task-specific variance (Gustavson et al., 2019; Miyake et al., 2000). However, complex batteries may feature an excessive abundance of fluency tasks (Aita et al., 2019; Patt et al., 2018), especially if the phonemic and the semantic variants are combined for the final analysis (e.g., Patt et al., 2018).

Moreover, the “executive—frontal—fluency” association is not only methodologically dubious but also inexhaustive. Though repeatedly pointed out throughout history, the verbal component is frequently overlooked or inadequately accounted for, in Thurstone's battery, one of the Vocabulary subtests loaded on the word fluency factor, which was interpreted as a proof of fluency being purely verbal (Fruchter, 1948). These days, such findings could suggest a deeper involvement of semantics in the supposedly desemanticized “fluency”. The seminal neuropsychological studies of cortical lesions mentioned a

“rather generalized higher-level language impairment” (Benton, 1968; Milner, 1964),

and fluency tests were supposed to tap into the “inner language” (Benton, 1967)—a strictly non-Thurstonian interpretation. As a result, the first aphasia battery incorporated a fluency test, although aphasic patients were deliberately excluded from some early studies (Benton, 1968). Some recent studies highlight the role of language ability (Lebkuecher et al., 2021; Whiteside et al., 2016) or verbal intelligence (Kraan et al., 2013) rather than executive control.

An overview of studies that attempt to coordinate PVF with executive or language ability is given in Supplementary Table 1. The table covers major executive tests that were selected according to several sources. Keyword search *via* Google Scholar was used, spanning the last decade (2015–2025), with a single pertinent article published in 2026 added. A more detailed description of the selection criteria and methods in given in Supplementary materials. The number of studies is relatively high, yet their comparability is dubious due to the diversity of paradigms, that is, specific tests may represent either “executive functions” or “language”. However, a broad range of tests within the study minimizes the effects of task impurity and thus remains methodologically inevitable. Even within a single test, different parameters are sometimes chosen and are to be born in mind while interpreting the conclusions (e.g., a statistical RT parameter vs. total number of words in confrontation naming). Approaches to dealing with data vary as well, such as considering semantic and phonemic fluency separately or together, or analyzing mixed participant groups. As the table shows, tests that may be separated by decades tend to be administered together. A testing paradigm often preceded interpretations and was later re-interpreted within the prevailing line of thought, thus undermining construct validity. Most tests were created prior to the key advances in neuroscience, hence the established neuroanatomical correlations are dubious. Apparently, most tests are generally sensitive to the presence of damage, yet their sensitivity and specificity might be overrated. Studies that administer them alongside fluency tests feature diverse demographics: some groups are rarely represented, while for others the findings are controversial. The current norms may be limited, dated or poorly compatible with more recent neuropsychological trends. Specific test instructions, variants of the paradigm, practice effects and emotional strain are rarely accounted for, lowering cross-study comparability. Overall, both “heritage approaches”—that is classical executive tests and the use of the total score in phonemic fluency—seem to yield inconclusive results yet crucial for disentangling the overall complexity of the theoretical constructs in question. In the final analysis, both terms, *fluency* and *executive*, seem to represent just the tip of the iceberg. The submerged part consists of variable and multifaceted theoretical constructs with several layers of tradition. New executive theories tried to provide a heuristic tool that could increase its explanatory power—while stemming from different sources: cognitive, clinical, behavioral or neural (Demetriou et al., 2019). Yet in case of fluency, it seems to further complicate the matter. It is unclear which constructs PVF as an “executive task” taps into, and how compatible they may be. As we have shown, fluency was originally linked to the frontal lobes and only subsequently to executive functions, which were not even clearly operationalized at the time. The connection between the frontal lobes and the executive construct has been debated (Alvarez and Emory, 2006; Stuss and Alexander, 2000).

To reiterate, the tripartite “executive/frontal/fluency” chain has accumulated a number of significant challenges, such as inherent complexity, lack of consensus on defining the functions, methodological circularity in test validation, the questionable ecological validity of fluency, and the persistent evidence for a substantial linguistic component. These challenges could be mitigated through evidence from neuroscience, as indeed suggested by some authors (Bunge, 2024). We review the evidence from this field in the following part.

## 4 Diving deeper into the brain using methods of neuroscience

In 1887, Albert Fournier famously stated:

“[s]peech is the only window through which the physiologist can view the cerebral life.”

**Figure 2:**
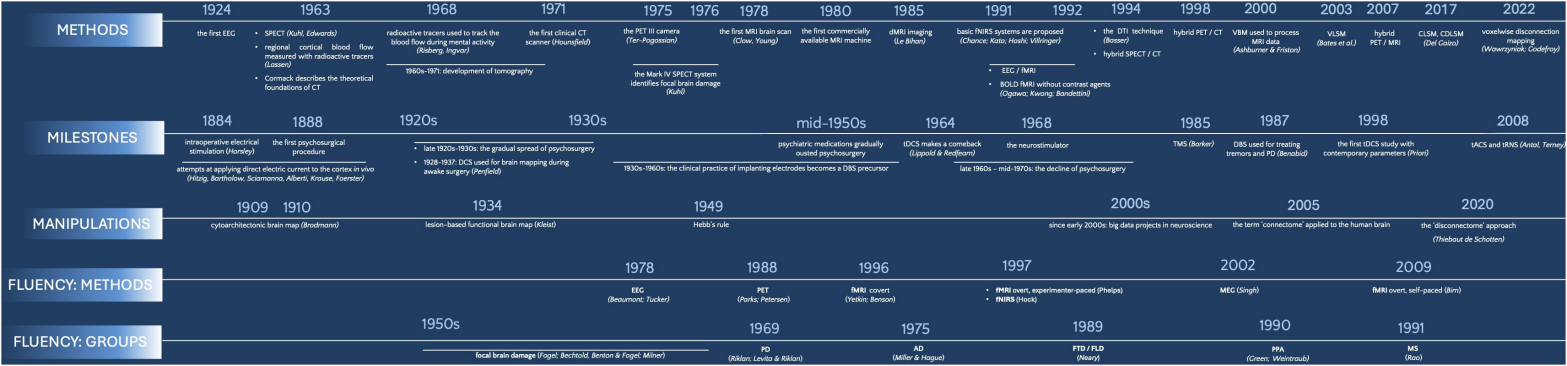
The evolution of phonemic fluency research technique along the development of neuroscience. Horizontal lines below the timeline indicate time periods.

For decades, this statement remained true for clinical studies, where neuropsychological tests supported by lesion data were often the only insight into neural processes. Early researchers who began shaping and applying the PVF test had limited knowledge about the brain as their studies were based on small-scale cohorts with extreme population bias (e.g., younger patients with traumatic brain injury or schizophrenia cases: Luria, 1966b; Milner, 2006). Lesion etiology was not accounted for as a variable (Bechtoldt et al., 1962; Fogel, 1962, 1960; Milner, 1964). Neuroimaging methods *in vivo* were frequently invasive, if feasible at all (Figure 2).

The subsequent evolution of neuroimaging methods has since provided a vastly detailed view of cerebral structure and function, from the micro- (e.g., tissue integrity, neuroplasticity, and metabolic processes) to the macroscale: white matter pathways connecting cortical and subcortical gray matter areas, into distributed networks and, ultimately, the “connectome” (Herbet and Duffau, 2020; Kowalczyk et al., 2022; Stevens, 2016; Stoecklein et al., 2020; Zink et al., 2021). Large studies (Biesbroek et al., 2021; Godefroy et al., 2024; Vonk et al., 2019) have improved control over such variables as age and etiology. Functions are now seen as supported by complex networks rather than isolated regions; within the latter, fine-grained differentiation has revealed more intricate connectivity patterns. In contrast to early works, current studies recognize the interplay between locus and etiology (Harris et al., 2022; Li et al., 2017; Schulz et al., 2022), as well as between gray and white matter damage in clinical cases (Bonilha et al., 2014; De Santis et al., 2019; Kao et al., 2019; Maitre et al., 2023; Zhou et al., 2022b). Method sensitivity has also become a controlled variable, and multimodal studies are used to achieve equilibrium (D'Esposito et al., 2003; Karanasiou, 2012; Varangis et al., 2021). These advances have propelled neuropsychology—including the study of speech, language, and executive functions—far beyond the tools available to pioneers like B. Milner and A. Benton.

This progress reveals a critical flaw in earlier work: many confounding factors could not be accounted for at the time (Kolb, 2022; Milner, 2006). Alzheimer's disease, Parkinson's diseaseand other aging-associated conditions often assessed with quick paradigms such as PVF, or investigated with other executive tests due to presumed frontal damage, actually yield combined effects of more widespread degradation. Known focal lesions have also been shown to cause widespread secondary damage or connectivity changes in remote areas (stroke, TBI: Stuckey et al., 2021; Thapa et al., 2021; aggressive gliomas: Stoecklein et al., 2020). In light of this network-based complexity, the “(pre)frontal—executive—fluency” chain appears fundamentally inadequate.

This inadequacy becomes clear when we examine its first element of the chain: the prefrontal cortex. Various versions of the “executive network”, which have emerged over the last decades, are not compatible with a single “executive” component and instead spread to other areas such as the parietal cortex. Some models postulate two types of control, “stable” and “adaptive”, which are particularly suitable for the fluency tasks (Dosenbach et al., 2007). More detailed frameworks integrate them into a multi-network system along with other functions (switching, processing input etc.), each involving certain prefrontal areas—which, in turn, feature their own “connectional fingerprints” to other brain regions. While proper interpretation is still pending, narrow “region-function” mapping does not appear feasible either at the level of the whole network or its components (Ardila et al., 2017; Buckner et al., 2008; Dixon et al., 2018; Fuster, 2015; Menon, 2011; Menon and D'Esposito, 2022; Menon and Uddin, 2010; Niendam et al., 2012; Stuss, 2011; Volle et al., 2012).

Instead of the traditional single entity, the prefrontal foundation of the model is increasingly seen as a complex of specialized regions. Consequently, the “executive” component of the tripartite model also begins to fragment as well. Cognitive models may be supplemented with neural evidence, as in the case with “hot/cold functions” (Salehinejad et al., 2021) or the classical “unity and diversity”, where verbal fluency may be reinterpreted as an activation-eliciting task (Rodríguez-Nieto et al., 2022). However, the “top-down” approach—that is coupling up-to-date physiological findings with the existing theoretical constructs—may overlook the functions outside the “canonical” executive scope (Bunge, 2024). Similarly, age-appropriate models at both ends of the lifespan should account for the recently discovered effects: network restructuring (differentiation in either development or aging), engagement of compensatory networks, changes in tissue structure and even lateralization (Cabeza, 2002; Cabeza et al., 2002; Edde et al., 2020; Reuter-Lorenz and Park, 2014; Schulz et al., 2022). In the rapidly aging world where educational achievement is prioritized, there is a growing need for valid, neurologically compelling cognitive assessment—including PVF as an easily accessible measure.

Naturally, the third link in the “(pre)frontal—executive—fluency” chain does not stay immutable either. Its association with the frontal cortex remains significant, but not localized (Baldo et al., 2010, 2006; Henry and Crawford, 2004c; but see Collée et al., 2023; Chouiter et al., 2016). Lateralization remains debatable too: left-lateralized findings are more typical, yet some studies evidence bilateral or right-lateralized patterns, which may—although do not necessarily have to—be explained by aging (Fonseca et al., 2021; Marsolais et al., 2015; Meinzer et al., 2009). Similar to “executive network(s)”, there are partially overlapping “fluency networks”, semantic and phonemic, which spread to white matter tracts and subcortical structures (Ellfolk et al., 2014; Glikmann-Johnston et al., 2015; Jossinger et al., 2024; Silveri, 2021; Won et al., 2021). Extracortical findings are clearly relevant to the status of PVF as an aspect of executive control, as the effect of subcortical lesions in PVF is comparable to the frontal ones (Andrade et al., 2012; Cipolotti et al., 2021). The cerebellum and the basal ganglia are apparently responsible for cognitive, language and emotional control within the executive system (Caligiore et al., 2017; Ide and Chiang-shan, 2011; Massara et al., 2025). The cerebellum seems implicated in PVF performance as well, while for the basal ganglia and the subthalamic nucleus the findings are inconclusive (Ellfolk et al., 2014; Grönholm-Nyman et al., 2025; Leggio et al., 2000; Silveri, 2021; Schlösser et al., 1998; Schweizer et al., 2010).

Ultimately, the link “(pre)frontal—executive” and the “(pre)frontal—fluency” seems more ambiguous than previously expected. The sheer complexity of the PVF task challenges direct mapping, especially considering the multiple neurophysiological and neuroanatomical dimensions, which may or may not influence performance (Shekari and Nozari, 2023). A clearer picture of fluency processes must include the language component. We will follow the step-by-step procedure previously used with executive functions and fluency: proceeding from the concepts operationalized within this level to the neural mechanisms. To this end, linguistic theories will be required no less than neuropsychological ones. Their development since the early 20th century may clarify not only the historical but also conceptual roots of some assumptions underpinning PVF as, first and foremost, a verbal task.

## 5 Putting the “verbal” back into “phonemic verbal fluency”

The classic “executive functions vs. language” dichotomy seems clear enough until a definition of “language” is required. The use of language in a PVF test is specific and complex, and three basic methodological paradigms approach it from different standpoints (Hickok, 2014; Perret et al., 2012). Linguistics views language as a coherent abstract system; psycholinguistics treats it as a process and behavior through experimental techniques, which subsequently form the basis for conceptual models; neurolinguistics and neuroscience of language focus on the instrumentally registered neural mechanisms that underlie these models. While all three can be organized into a hierarchy, they are typically kept separate in research (Poeppel, 2012; Poeppel et al., 2012; Strijkers and Costa, 2016).

Each discipline offers a valuable perspective on PVF. The lexical units in the test are ordered according to the initial phonemes, which are, in turn, linked to a grapheme. Therefore, at least the phonological level must be clearly conceptualized. Content relatedness and morphological similarity, too, are involved in rule-based list generation, which means that lexis and grammar also contribute to task performance. Various psycholinguistic processes operate at each level, such as semantic priming; their neurophysiological underpinnings may be experimentally explored through previously outlined imaging techniques. Respective contributions of each discipline to interpreting VF tests are best viewed from an evolutionary perspective. It shows how theoretical views on language developed from structuralism to cognitivism through generativism, getting influenced by psycholinguistic models—with or without neural correlates.

The first fluency tests emerged during the heyday of the structuralist approach to language studies. Form was supposed to dominate over meaning, with no links established between language and thought. The lexicon was generally dismissed as an “appendix of the grammar”, for instance, in Bloomfield's works rejecting mentalism (Bloomfield, 1933). Striving to free linguistics from subjectivity—to treat it as a science rather than art—this descriptive and partially mechanistic approach was further adopted by neo-Bloomfieldian structuralism, for example, in the works of Z. Harris and F. W. Harwood (Léon, 2021; Tomalin, 2006). This legacy persists in clinical fluency scoring, which often disregards semantic or morphological strategies. For example, listing derivatives (“farm”—“farmer”) or words belonging to the same part of speech (“fabulous”—“famous”) was for a long time considered a rule break, and thus removed from analysis, dismissing the potential insights into the cognitive-linguistic interplay. Generativism, too, generally focused on syntax at the expense of lexis (Chomsky, 1957; Chomsky and Halle, 1968). Only the “Lexicalist Hypothesis” (Chomsky et al., 1970) finally acknowledged the lexicon as a separate module—consisting of units with semantic, syntactic and, sometimes, phonological features. This paved the way for functional and cognitive approaches to language (Jackendoff, 1997; Pustejovsky, 1998); however, by this time the PVF paradigm had long existed within the clinical domain, with its theoretical foundations dating back much further.

Hence, a paradox emerges: the PVF test is built on a strict division between form and meaning dating back to early 20th-century linguistic theories, which are largely considered outdated, yet it remains in use. Generativism may have enriched it indirectly with a series of useful oppositions: linguistic meaning required for sentence generation opposed to the conceptual (encyclopedic) one (Evans, 2009; Paradis, 2003); “competence” (knowledge of language) and “performance” (its actual use); or syntax and morphology (in lexicalism). However, this level of detail was achieved by fluency research much later, with the arrival of cognitive approaches. Only by the start of the 21st century did context and then semantics fully re-enter the field, restoring the long-neglected link between the structure of conceptual categories and their epistemological function embodied in language (Geeraerts, 1993).

Due to this lag, normal language processes during test performance may have been misinterpreted as signs of executive failure. For example, phonological cues in the fluency task (e.g., /f/) activate lexical-semantic networks (“fish,” “fishing,” and “fisherman”), blurring the line between “phonemic” and semantic patterns. If ignored, this link may be attributed to a stronger executive deficit than is actually present (if at all), since the task requires overriding language processing mechanisms to a considerable extent. Even without violating the rules, participants often cluster words by semantic fields (e.g., “field,” “farmer,” “fruits” for F) or derivational morphology (“run,” “runner”). Semantic likeness and derivational-morphological strategies beyond phonology are cognitively normal but still ignored by scoring practice, which has mostly fossilized in its “structuralist” shape.

Similar to other methodological frameworks of the 20th century, this scoring approach is inherently modular. By the middle of the century, language has been viewed as a system of independent modules, linked through interfaces—seen as an innate module autonomous from other cognitive structures (in the generativist paradigm: Chomsky, 1986; Fodor, 1983). The prominence of generativism must have reinforced hierarchical modularity, and so did the breakthroughs in computer science and the “automatic turn” of the 1940s−1960s. The military applications of linguistics, such as cryptanalysis, translation, and language teaching in the army (Léon, 2021). A helped formalized language as a modular structure comprising distinct levels, with operations performed on each level-specific unit—an essentially algorithmic model promoted by the computer metaphor. Since then, the key trends have remained, such as the later revision of generativism: the Minimalist Program (Chomsky, 1993), where language modules are strictly encapsulated and do not allow interference from other cognitive systems once within-module computation starts (Newell and Sailor, 2023). The resulting independence of phonology, morphology, and semantics, however theoretical, fully fits the standard clinical interpretation of PVF (see Supplementary Table 1, Part 2).

Unlike language theory, psycholinguistics has been concerned with mental operations from the start. Psycholinguistic models were constructed on clinical evidence of specific language aspects being impaired yet others spared; isolated speech errors in healthy speakers also pointed toward modularity (Dell, 1986; Fromkin, 1987, 1973, 1971; Garrett, 1975). In the 1960s, the “mental lexicon” was postulated as a separate level of language representation in the mind (the term introduced in: Treisman, 1961). Different language levels (semantic, lexical, grammatical, orthographic, phonological etc.) could be conceptualized as “lexicons” in their own right: modules where specific information is stored and level-specific operations are performed (Austin, n.d.; De Sousa and Gabriel, 2015). As a result, word retrieval—for example, during list generation on a fluency task—was conceptualized as a stepwise progression through isolated stages, where each could start only when the previous was completed. Despite certain reinterpretations (to be discussed later), the concept of the “mental lexicon” remains a staple of psycholinguistics.

Thus, similar processes can be observed both in linguistics and psycholinguistics. While generativism is typically opposed to cognitive linguistics, it sought for the mental basis of language as opposed to external behavioral factors, and the mentalist approach to grammar as universal and inborn has been widely recognized as instrumental in the “cognitive turn” (e.g., Bolhuis et al., 2023; Chomsky, 1959). The generativist “linguistic competence” presumed a lexical module to store linguistic meanings, and the debate surrounding the “mental lexicon” enabled the later search for cognitive patterns behind lexis. Still the dynamic component of “performance”—the actual processes of perception and production—were to be explained.

Another key concept rooted in 20th-century psycholinguistics is spreading activation (Collins and Loftus, 1975)—a model typically used to explain PVF performance, even in more nuanced studies that go beyond the scope of the present paper (e.g., Anzak et al., 2011; Ross et al., 2007; Zabberoni et al., 2017). Its predecessors were hierarchical, probably due to the computer metaphor, for example, the “logogen”—a hypothetical interface between the conceptual and phonological levels (Morton, 1969); or the hierarchical network model of semantic memory (Collins and Quillian, 1969; Quillian, 1967). In contrast, spreading activation was intended to be non-hierarchical, but soon followed the trend, followed by models of lexical access (e.g., Dell, 1988, 1986; Fromkin, 1971; Garrett, 1975; Roelofs, 1992). The eponymous “activation” spread through the network of the mental lexicon from semantic to lexical to phonological nodes. However, modularity allowed little cross-level interaction—so a lemma arrived: essentially an interface node between concepts and phonology (like the logogen) that comprises morphological and semantic features (Kempen and Hoenkamp, 1987; Levelt et al., 1998).

These models were practical, easy to apply and visualize in a “box-and-arrow” shape: with information flowing sequentially (arrows) between distinct modules (boxes), each of which is effectively a black box, not transparent to the researcher (Bullinaria, 2003). With clearly separate verbal levels, “box-and-arrow” frameworks are convenient for error-based clinical assessment relying on verbal paradigms such as PVF (e.g., Vonberg et al., 2014; for another popular testing paradigm, see German, 2015; German and Newman, 2004). For instance, better performance on the phonemic rather than semantic fluency task in AD supposedly indicated the independence of the conceptual level from phonology—and a dictionary-like alphabetic index in the mind (Bayles et al., 1989). A task based on the intentional split between language levels was bound to be represented in a “box-and-arrow” form, even at the cost of adequately representing complex linguistic processes. Ever since, modularity has prevailed in clinical as well as theoretical PVF research, especially involving the “mental lexicon” as a storage block: referenced in a generic way (Juhasz et al., 2012) or confined to a specific level (the “phonological mental lexicon”, particularly in bilinguals: Kassir et al., 2025; Neergaard et al., 2019, 2022). PVF performance is typically conceptualized as cued search within a closed language module, even if the lexical access model is not discussed consistently (with the rare theoretically detailed exception of Juhasz et al., 2012; see Bose et al., 2022; Lindsay et al., 2021; Mengisidou and Balladares, 2023; Park et al., 2022; Pekkala et al., 2013; Vonberg et al., 2014). The search, too, is conceptualized as sequential, hierarchically proceeding from one closed module to another (Park et al., 2022; Vonberg et al., 2014).

Therefore, despite the popularity and a strong explanatory power of classical models, a question arises: could alternative theoretical proposals be integrated with PVF? Several such attempts will be outlined below.

## 6 Beyond box-and-arrow: outlining network models and embracing the complexity of verbal fluency

The previously mentioned computer metaphor may have inspired both non-hierarchical and hierarchical models. The “mind-as-computer” approach, which dominated theoretical thought in early cognitive science, was clearly modular and hierarchical (Figdor, 2017; Léon, 2021). Yet, on the other hand, parallel processing in computer science and information theory became feasible rather early (e.g., Smith, 1952) and may have provided a welcome metaphorical alternative. Numerous terms used in the well-described theories do stem from information technology: “nodes” and “networks” in spreading activation, or even “neuron” as a computational unit (cf.: McCulloch and Pitts, 1943; Rosenblatt, 1958). Network theory, which is now increasingly applied across various fields, including cognitive studies, could be the most recent offshoot of the computer metaphor (Ferguson and Foley, 2024; Min et al., 2025; Stella et al., 2024).

While the original non-hierarchical spreading activation model postulated bidirectional links between interconnected nodes, unidirectional lexical access theories allowed only the spread of activation from concept to lemma, then proceed to phonology, phonetics and articulation. “Parallel” accounts, which retained bidirectional links, were proposed for sensory processing, then extended to cognitive operations and specifically language perception (McClelland and Elman, 1986; McClelland and Rumelhart, 1981). By the late 20th century, after intense debates they entered both language theory and psycholinguistics (Rogers and McClelland, 2014; Townsend, 1990). In the parallel distributed processing (PDP), or connectionist, theory neurons were supposed to operate simultaneously in a network echoing neural mechanisms, such as Hebbian learning (Rumelhart and McClelland, 1986). In language theory, the late-1990s Parallel Architecture within generativism (Jackendoff, 2002, 1997) described the co-existence of conceptual/semantic, syntactic, and phonological representations that follow independent rules. Cognitive frameworks embraced parallelism quite eagerly: see Halliday's concept of “lexicogrammar”, which stressed the continuity between and indivisibility of grammar and lexis (Halliday, 1961); R. Langacker's cognitive grammar, which blurred the line between lexicon and syntax (Langacker, 1991, 1987); L. Talmy's works on semantics of spatial relations in grammar ensuring the link between semantic structure and lexical, morphological, and syntactic structure embedded in discourse (Talmy, 2000, 1985); A. Goldberg's concept of construction grammar and the constructionist approach in the tradition of cognitive linguistics with semantics at its core (Goldberg, 2013; Kay, 2022). In psycholinguistics, more interactive, bi-directional models of lexical access (Dell, 1986; Dell and O'Seaghdha, 1992) initially lost the battle for popularity but remained methodologically compelling—especially for fluency, with its inevitable interaction between the “desirable” phonology and the “undesirable” lexico-semantic component. As was the case with executive functions, the debate between serial and parallel accounts could be resolved by turning to biological evidence, that is, neuroscience of language.

The classic early 20th-century “Broca-Wernicke-Lichtheim-Geschwind” model of language is functionally modular and spatially limited to a set of cortical areas. Though neatly matching modular language theories (De Sousa and Gabriel, 2015), it does not fully agree with current neuroscience as described above. Alternatives are less linear, more distributed, more aware of neurofunctional mechanisms and extracortical involvement. The dual-stream architecture distinguishes between two functional routes: the ventral stream maps sound onto meaning, and the dorsal one maps it onto articulatory representations, thus carrying out sensory-motor integration (Hickok and Poeppel, 2007, 2004, 2000; Saur et al., 2008). Originally limited to perception, it later assimilated production mechanisms as well (Hickok, 2012). Eventually it has been adapted to written language processing—a matter of interest to PVF, which is letter-based rather than phoneme-based (Kearns et al., 2019). Another model, the core language network, differentiates between crucial language processing areas and secondary ones that support non-language operations according to task demands (Hagoort, 2017; Hertrich et al., 2020; Rocca et al., 2020). In the Hebbian cell assembly model, unlike these two, various regions are initially activated almost simultaneously during speech processing (e.g., Pulvermüller, 1996; Pulvermüller and Preissl, 1991; Strijkers and Costa, 2016).

Lexical access as a domain of fluency research can be explained within most of these models. The rule-based separation of lexis and phonology in PVF shifts attention to the involvement of lexical vs. phonological language levels. The dual-stream architecture, for instance, has produced a hierarchical feedback state control (HFSC) model (Hickok, 2014, 2012), which highlights the role of motor control in phonological processing and keeps lexical activation separate. A dual lexicon model matches two types of word representations to the two streams (Gow, 2012). Both models are relatively modular, as different layers of processing are mapped to specific areas. By contrast, in parallel processing there is no specific lexical layer: semantics and phonetics are co-activated and bound in a word assembly *via* Hebbian learning (Kerr et al., 2023; Strijkers, 2016; Strijkers and Costa, 2016). Consequently, phonological/phonetic activation might happen early, which has been predicted by theoretical PDP models postulating a non-hierarchical network of links between word properties (Seidenberg and McClelland, 1989), followed by the no-lexicon proposal in psycholinguistics (Elman, 2004).

Meanwhile, PVF research has not fully adopted alternative theories. Some recent works reference the dual-stream model (Mousavi et al., 2020; Rodriguez-Porcel et al., 2021) or compare theoretical models with neural findings (although fluency maybe merely a control task: Migliaccio et al., 2016). Concept-driven neural models may be referenced too, along with other models, in studies that involve fluency in some quality (Tao et al., 2020; Verfaillie et al., 2019). Despite the theoretical appeal of these interactive models, PVF research remains largely anchored to modular and hierarchical cognitive models (e.g., Vonberg et al., 2014) for two major reasons.

First, the status of alternative models is ambiguous. The no-lexicon approach, for instance, is grounded in discussions about the conceptual foundations of mental lexicon models (De Sousa and Gabriel, 2015; Libben, 2022, 2017)—yet other studies indicate isolated processing areas for each language level, supporting modularity and the lemma concept (Wilson et al., 2018). Some studies explicitly map language components to regions (e.g., syntax: Grodzinsky et al., 2021; Matchin, 2023; Matchin and Hickok, 2020; Pylkkänen, 2019; however, a more distributed view of syntax is discussed in Fedorenko et al., 2020; Shain et al., 2024). While most theories of cognitive functioning reject one-to-one mapping (MacKay-Brandt, 2011; Strijkers, 2016), some areas demonstrate an obvious domination even in distributed models. Whether primary or integrative, bottom-up neurolinguistic models that back up an existing construct with physiological data may rely on mere correlations, lacking explanatory power and conceptual finesse. Novel models are still a subject of debate: see Carota et al. (2023), Kerr et al. (2023), Indefrey (2016), Munding et al. (2015). Combining them with an already problematic fluency task would imply a double experiment. At the same time, conventional psycholinguistic models have received some neurophysiological backing and are no longer “disembodied” (e.g., Damasio et al., 1996; Dell et al., 2013; Indefrey and Levelt, 2004).

Second, the modularity of PVF may be task-specific. Its constrained design, including time limits and phonetic cues, compartmentalizes lexical access into discrete stages: first-letter search, inhibition of semantic competitors, cluster switching. This approach aligns with serial “box-and-arrow” frameworks. However, naturalistic language production relies on parallel, interactive processes: semantic, phonological, and syntactic cues co-activate dynamically, as evidenced by tip-of-the-tongue states and semantic intrusions in phonemic tasks. As a result, neuroscientific studies reveal discrepant patterns of PVF vs. natural speech. While fluency tasks activate seemingly modular dorsal/ventral streams (Rodriguez-Porcel et al., 2021), natural speech engages distributed networks (Cai et al., 2025; Hagoort, 2017; Zhou et al., 2022a). This duality could make more naturalistic models unsuitable for fluency, at the same time calling for differentiation between artificial task constraints such as phoneme rules and natural retrieval processes such as semantic-derivational clustering, rather than juxtaposing them, especially in fluency scoring.

Neural correlates appear questionable due to the sheer complexity of PVF. In the distributed, network-based neuroscientific framework, simultaneous executive, and language processing implies shared correlates: the areas, such as prefrontal, temporal, and parietal cortex, subcortical areas and various white matter tracts (e.g., Hertrich et al., 2020; Tao et al., 2020). Some of these findings are methodologically safe: take the involvement of the temporal lobe, which fits almost all neurolinguistic models, attests only to the obvious fact that verbal fluency is *verbal*, and hence recruits semantics to an extent. Considerably more confusing are the areas implicated in multiple functions, such as subcortical ones. The cerebellum is involved in executive (Aiello et al., 2024; Cao et al., 2021; Chen et al., 2020) as well as language control (the “lateralized linguistic cerebellum”: Mariën et al., 2001). The pathways that link it to the thalamus and the cerebral cortex (Habas, 2021) play a distinct role in PVF in healthy younger adults (Jossinger et al., 2024). As a result, it is impossible to state whether the contribution of subcortical areas to fluency is due to language processing or some other set of higher functions. Additionally, there are multiple confounding variables: age, pathology, method sensitivity, and their combinations. Thus, lateralization in fluency could be affected by changing patterns in old age or a method insensitive to lateralization, such as rs-fMRI (Ahmed et al., 2023; Kumar et al., 2020). On top of that, methodological flaws are added: the lack of healthy controls (Carbone et al., 2023), overlooked confounding variables (e.g., presence of aphasia: Li et al., 2017) or co-occurring non-cognitive symptoms (such as dysarthria: Corben et al., 2024). Not surprisingly, neural findings for fluency remain largely inconclusive.

In summary, the interpretation of PVF is trapped between a rich but outdated modular theory and modern, interactive models that better reflect cognitive and neural reality. Neural data alone cannot resolve this stand-off due to the task's inherent complexity and methodological challenges. The optimal way to bridge the gap would be to explicitly acknowledge the task's complexity rather than seek for a single “correct” model. Disentangling the rule-based constraints from the underlying natural language capacity could enable revised scoring systems that credit, rather than penalize, semantic and morphological clustering as valid cognitive-linguistic strategies.

## 7 Discussion. On the way to the integrative interpretation of phonemic fluency

This bird's-eye view of the theoretical landscape of PVF research reveals a range of theoretical, methodological, and technological topographies that have taken shape since the test developed and implemented. It remains in use as a sensitive, quantifiable measure of *constrained lexical access under time pressure*, yet its potency requires proper interpretation of historical, theoretical and methodological contexts as a formative influence. From a present-day perspective, PVF involves distributed language and cognitive networks, particularly left-lateralized systems involving frontal, temporal, and subcortical regions, responsible for a range of behavioral and language functions. Its complex nature goes far beyond its status of a purely “executive” or “frontal” task. In this review, we therefore attempted to uncover any implicit assumptions that could influence the data interpretation.

The shared evolutionary pattern of the three disciplines we have considered—neuropsychology, neuroscience, and linguistics—centers on the modular/non-modular dichotomy. At some point, it turned into a continuum, along which the respective theories were positioned. In linguistics, interacting but distinct levels of language—phonology, lexical-semantics, and syntax—are intuitively perceived as modular, and the most influential 20th-century frameworks conceptualized them accordingly. Psycholinguistics investigated the processing mechanisms underlying each level; neuropsychology, in its turn, sought for their neural foundations, which sometimes overlapped with more general behavioral and cognitive processes. Due to its more objective physical nature, neuroscience has long been expected to settle the debate. However, neuroimaging evidence attests to both modular and non-modular phenomena. Thus, cross-disciplinary unification might have been prompted by the pervasive mind-as-a-computer metaphor. Its later offshoot, mind-as-a-network may be driving present-day researchers to look for decentralized theoretical models. If this is true, the way forward requires an even more cautious approach to data interpretation—especially the complex multilayered verbal data that PVF provides.

In fluency research, modularity was postulated in early batteries of primary abilities, which split verbal ability into independent aspects: dealing with ideas and meanings (V-factor) and single and isolated words (W-factor: Thurstone, 1943, 1938). Consequently, the task lived on as a paradigm that separated language levels, later reinforced by the modularity of most 20th-century research. The current fluency paradigm, with separate semantic and phonetic fluency tasks, rests on these heritage assumptions, rarely (if ever) discussed in clinical practice (e.g., “box-and-arrow” models: German, 2015; German and Newman, 2004).

Considering the probable oversimplification, not misinterpretation: indeed, PVF is not as semantic-oriented as everyday speech (e.g., Mironets et al., 2023; Shao et al., 2014), yet clearly more so than Thurstone's word fluency. The monolithic executive/frontal/fluency approach must have developed through such simplification—even though early interpretations were mostly tentative. In neuroscience, for instance, even the earliest seminal works referred to the connections between brain areas, which at the time were out of researchers' reach (Tremblay and Dick, 2016); scholars of the 1950s−1960s did not attribute poor performance on neuropsychological tests to focal damage only. Neither did Binet and Simon intend their test to assess inborn abilities, or its components to be applied independently. Thurstone was equally cautious about the implications of his battery. Within 10 years from its release, its fluency construct was challenged, as additional types of fluency were suspected (Fruchter, 1948). All this proves that the evolution of theories does not necessarily proceed from simple to complex: it is merely more natural to temporarily discard the complexity that cannot be handled with current means and methods. With the current tools, capturing it finally seems feasible—at least for PVF.

The step-by-step analysis of linguistic, neuropsychological, and neuroscientific mechanisms of PVF fits the ongoing multidisciplinary trend. Once the possible “thinking pattern fallacies' are fully realized, a data-driven review of the existing neuropsychological tests seems necessary. One could revise and update their theoretical and methodological implications through neuroscientific evidence; draw data from open-access databanks to increase reliability; introduce computer-assisted test administration for more dynamic and flexible testing (Bilder and Reise, 2019). None of the historical steps undertaken in this study seems to have led to an unambiguous conclusion established once and for all: most likely because the PVF test is a prime example of a construct-driven assessment. Construct-driven tasks, as follows from their name, aim to measure an abstract construct through performance rather than study the actual performance (Burgess et al., 2006). The construct typically associated with PVF—executive functions—is ambiguous and changeable, as revealed by a closer look at the *executive/frontal/fluency* chain. Neuroscientific evidence, however detailed it may be, is mostly construct-biased—perhaps as much as the neuropsychological tests it is supposed to back up. As stated previously, neuroimaging correlates in task-based paradigms may be determined by the range of tests, which, in turn, reflect the chosen construct. This “vicious circle” explains the difference between mapping studies that apply alternative models of executive functions (i.e., the “hot/cool” one: Colautti et al., 2022; Salehinejad et al., 2021; LaBar and Cabeza, 2006; compare: Duncan and Friedman, 2024). With a data-driven, bottom-up approach, highly specified target variables would prevail over historically fluid constructs (Bilder and Reise, 2019): for example, a particular behavior (Holleman et al., 2020) or test properties in a given population (Suchy et al., 2024a). Still, the problem may reach further.

In fact, the three disciplines involved in verbal performance operate at different levels of abstraction. The links between them are correlational at best, and a link between electrophysiological cell activity, a particular behavior and a theoretically postulated language unit only attests to “the Flatland fallacy”—a simplified mode of thought that views correlation as explanation (e.g., Jolly and Chang, 2019). An actual causal link requires a common basis: probably computational, as the basic premises of cognitive science are mathematical and logical (Figdor, 2017; Jackendoff, 2025; Martin, 2020; Parsons and Duffield, 2020; Poeppel, 2012). Without it, a “bottom-up” approach in neuroscience of PVF would cause further confusion (Bilder and Reise, 2019). Fora computational model of PVF, the underlying processes must be clearly conceptualized. Hence, for the time being it would be sufficient to ask a single question: how to make the PVF paradigm more informative?

The metaphorical Rosetta stone seems to be in the third link in the executive/(pre)frontal/*verbal* fluency trio. Most studies mentioned in this review provide a single fluency measure: the total word count. The mechanisms of word production under restricted conditions, however, are too complex to be captured by it. To enhance the research utility of the test, it is necessary to incorporate more nuanced item-level or temporal metrics (e.g., clustering/switching, initial latency, pauses, word frequency, age of acquisition etc.), which could reflect various underlying processes better. The most widely used metrics beyond the total score are clustering and switching (Troyer et al., 1997). Theyhave also been treated in various ways (Bushnell et al., 2022; Lundin et al., 2023; Park et al., 2022; Vonberg et al., 2014), and completely novel metrics have been proposed (Sapunova et al., 2025). Importantly, the third link in the “executive/(pre)frontal/verbal fluency” chain is the least explored—and in this respect free from layers and layers of neuropsychological heritage that we have been trying to disentangle so far.

The studies that have attempted this approach offer scarce, yet promising conclusions. Linguistic item-level metrics such as lexical frequency have proven sensitive to svPPA (Delgado-Álvarez et al., 2021; Henderson et al., 2023) and MCI (Dekhtyar et al., 2024). Psycholinguistic measures such as familiarity may differentiate between subtypes of cognitive impairment (Rofes et al., 2019); inter-item measures, especially temporal ones, could indicate its presence (Jacobs et al., 2021). This may seem insufficient for clinical use (see Henderson et al., 2023). Still, they may reveal latent cognitive and language mechanism, as shown by their relation to various demographic parameters, each other and the total score (Cho et al., 2021; González-Nosti et al., 2024). Despite the scarcity of neuroimaging studies that employ item-level metrics, some links have been established. For instance, those between word generation rate (rather than the total score), frontal gray matter volume in elderly adults (Pace et al., 2023) and the type of damage (Cipolotti et al., 2021), orinter-word intervals and white matter integrity in both healthy subjects and patients (Rodríguez-Aranda et al., 2016). In Sun et al. (2022), transcranial alternating current stimulation of the prefrontal cortex shortened response times in the initial segment of the trial; and STN-DBS may modify fluency parameters without affecting the total score (Grover et al., 2019).

With the arrival of novel parameters, better understanding of fluency mechanisms may be reached (Alhanai et al., 2017; Au et al., 2017; Diaz-Orueta et al., 2020; Sapunova et al., 2025). Complex and multifactorial as it is, fluency performance seems to require more specific and varied measures of core language ability with sufficient construct validity (e.g., naming, vocabulary, and vocabulary size) and specific executive functions. Ideally, these measures should be broken down into equally detailed metrics to ensure that granularity of data increases uniformly across all measures.

An overview of the PVF paradigm in its historical context reveals multiple influences, shifts and problematic points to manage. By shifting focus from the ambiguous total score to the rich data embedded within the response patterns, the PVF test can evolve beyond its historical constraints to yield more precise and meaningful insights into the complex interplay of language and cognition.
