# Supplementary material for: The evolution of phonemic verbal fluency test: bridging tradition with contemporary insights
Source: Front Psychol. 2026 Jul 17;17:1774256. doi: 10.3389/fpsyg.2026.1774256 (PMC13424034; doi:10.3389/fpsyg.2026.1774256)
Supplement: Supplementary file 1 [file Data_Sheet_1.pdf]

Supplementary Material

Methods and Criteria

Seven major executive tests were selected according to their popularity, as listed in: Faria et al., 2015; Baggetta and Alexander, 2016; Ardila and Ostrosky, 2022. The search was conducted via Google Scholar, using the keywords ‘phonemic fluency’ and ‘Stroop’ / ‘digit span’ / ‘N-back’ / ‘Go / No-Go’ (a separate query for several spelling variants) / ‘Trail Making Test’, ‘TMT’ / ‘Tower of Hanoi’, ‘Tower of London’, ‘WCST’ respectively. The ‘Tower of Hanoi’ query yielded no matches, and the ‘Tower of London’ was used instead. Only the works published in the last decade (2015—2025) were included; an overview of earlier articles that drew correlations between executive tests and phonemic fluency can be found in Kraan et al., 2013. As an exception, several earlier research articles were added on the basis of their prominence, as reflected by the citation count (in: Villalobos et al., 2023). For the language tests, only the time scope criterion was applied. For the sake of ‘brevity’, ‘language’ was chosen as an umbrella term to encompass various aspects of language functioning explored in respective works. Reviews and metaanalyses were not included, as well as the studies that listed the tests in question separately without attempting to link them to the performance on VF tests by any method.

Table 1

Part 1. Executive Functions

Test & Theoretical construct	Limitations	Correlations with the overall score discovered	Correlations absent or not significant	Phonemic Fluency Implications
**Stroop test** (Stroop, 1935) inhibition	Incomplete cross-study comparability due to different test variants (Giovannoli et al., 2020); normative data for some versions of the test based on small or demographically incomplete samples (Howieson, 2019)	healthy adults (González-Burgos et al., 2019; González-Burgos et al., 2021); MS (Delgado-Álvarez et al., 2021); STN-DBS in PD (Houvenaghel et al., 2018); stroke survivors (Turunen et al., 2016); bilinguals with aphasia (Patra et al., 2020a)	healthy adults (Stolwyk et al., 2015; Turunen et al., 2016; Patra et al., 2020a, b — bilinguals and monolinguals; Svindt et al., 2023); adults with RRMS (Svindt et al., 2023)	The potential shared neural substrate — principally left inferior frontal gyrus and anterior cingulate cortex — may explain the correlations in clinical populations whose inhibitory resources are genuinely depleted, while ceiling effects mask the relationship in healthy adults. Future research should specify the exact Stroop variant used while controlling for processing speed as a potential confound.

**Digit span** (Galton, 1887; Jacobs, 1887 working memory (backward), attention (forward)	Possibly not sufficiently difficult (Patra et al., 2020a) or sensitive in mixed samples (Egeland et al. 2026); sensitive to brain damage but incapable of differentiating between frontal and non-frontal stroke (Tamez et al., 2011); not valid as an attention test despite common practice (Treviño et al., 2021); measures short-term memory rather than working memory (Diamond, 2013)	healthy adults (Turunen et al., 2016; González-Burgos et al., 2019, 2021; Kavé and Sapir-Yogev, 2020; Filippi et al., 2022 — monolinguals); healthy children (Filippi et al., 2022 — monolinguals); stroke survivors (Turunen et al., 2016); adults with MS (Delgado-Álvarez et al., 2021); adults with DS and unspecified ID (Stavroussi et al., 2016); deaf children with CI (De Giacomo et al., 2021); adults with PD (Galtier et al., 2017)	healthy adults (Stolwyk et al., 2015; Patra et al., 2020a, b — bilinguals and monolinguals; González-Burgos et al., 2019: late elderly; Filippi et al., 2022 — multilinguals; Ucheagwu et al., 2024 — community-dwelling older adults); healthy children (Kavé and Sapir-Yogev, 2020; Filippi et al. 2022 — multilinguals); bilinguals with aphasia (Patra et al., 2020a)	According to conventional models, PVF recruits the phonological loop to buffer recently generated words and prevent perseverative errors; so does digit span forward. In the working memory model, the backward condition is also supposed to engage the central executive, which oversees the strategic demands of fluency (initiating, monitoring, and switching subcategories).<br>Correlations are robust across healthy adults and most clinical groups but absent in multilinguals and older adults — populations where cross-linguistic management and compensatory retrieval strategies, respectively, introduce variance that overrides the phonological loop overlap. Future research might study forward and backward scores separately and include language history or proficiency as covariates in multilingual studies.
**N-back** (Kirchner, 1958; Mackworth, 1959)<br>working memory; attention	Does not reliably reflect inter-individual differences in WM (Jaeggi et al., 2010); doubtful correlations with other WM measures, implying questionable construct validity	healthy adults (Gajewski et al., 2018)	Duan et al., 2025	N-back and PF demand continuous updating of working memory contents and suppressing outdated representations (frequently linked to the construct of the central executive). However, N-back's contested construct validity severely limits the interpretability of any

	(Jaeggi et al., 2010; Kane et al., 2007); possibly a worse indicator of WM than other tasks such as complex span (Schmiedek et al., 2014)			correlation with fluency; the evidence is sparse, and the findings controversial.
**Go / No-Go** (Donders, 1868 (original); 1969 (English translation)<br><br>inhibition	Not sufficiently sensitive or specific as a diagnostic measure in psychiatric disorders (Wright et al., 2014); different task types activate different brain regions (Simmonds et al., 2008); doubtful construct validity according to neural data: fMRI activations are linked to higher attentional and working memory processes, not specifically inhibition (Criaud and Boulinguez, 2013); limited content validity: neuropharmacolo gical differences between stop-signal and Go / No-Go indicate different types of inhibition, despite some neural overlap (Eagle et al., 2008)	healthy younger children (Wen, 2019; Filippi et al., 2022 — multilinguals and monolinguals)	healthy older children and adolescents (Wen, 2019)	Although Go/No-Go nominally requires response inhibition — a construct also implicated in fluency — fMRI evidence links task performance to attentional and working memory networks rather than a dedicated inhibitory mechanism. Correlations with fluency therefore may reflect some shared attentional demands rather than true inhibitory overlap. In younger children, where cognitive control is developmentally undifferentiated and both tasks rely on general self-regulation. The motor-response format shares no surface or structural resemblance with the verbal-phonological demands of fluency, which may explain the consistent absence of correlations in adults.

**Tower of London** (Shallice, 1982) planning	Doubtful cross-study comparability due to the discrepant cognitive demands posed by different versions of the task (1982, 1997) or the effects of instructions and cueing conditions (Unterrainer et al., 2003; Unterrainer et al., 2004; Koppenol-Gonzalez et al., 2010); cognitive demands depend on the level of difficulty (Tyburski et al., 2021); planning as a construct measured by the test is poorly defined and operationalized (Koppenol-Gonzalez et al., 2010); the task measures other abilities such as fluid intelligence and visuospatial WM (Unterrainer et al., 2004; Zook et al., 2004; D'Antuono et al., 2017)	healthy children (Filippi et al., 2022 — multilinguals and monolinguals); adults with MS (Delgado-Álvarez et al., 2021)	deaf children with CI (De Giacomo et al., 2021)	Strategic planning and PVF require structuring output in advance and monitoring progress towards a goal. Correlations in MS patients and multilingual children suggest that when automated lexical search breaks down, explicit planning becomes more relevant to fluency performance. Failure to correlate in CI children may reflect a developmental dissociation between planning and phonological access. Critically, visuospatial WM and fluid intelligence co-vary strongly with ToL performance, so correlations with fluency may implicate the g-factor variance rather than planning only. Future research might partial out fluid intelligence and visuospatial WM before considering ToL-fluency correlation as evidence in favour of a planning-specific contribution.
**Trail Making Test** (TMT: Army Individual Test Battery, 1944)<br><br>inhibition; working	Sensitive to brain damage, especially in stroke and AD, but incapable of differentiating between frontal and non-frontal stroke (Tamez et al., 2011);	healthy adults (Kasper et al., 2015; Turunen et al., 2016; Barbosa et al., 2017; Aita et al., 2019; González-Burgos et al., 2021); stroke survivors (Turunen et al., 2016); adults	healthy adults (Patra et al., 2020a — bilingual; Ucheagwu et al., 2024); bilinguals with aphasia (Patra et al., 2020a); a mixed sample of children with SLI and healthy children (Henry et al., 2015)	TMT-B's set-shifting between alternating letter and number sequences parallels the strategic switching between phonological subcategories that underlies fluency performance. The broad correlational success of

memory; attention; switching	insufficient as an isolated measure of frontal dysfunction, as it requires a broadly distributed brain network (Chan et al., 2015); TMT-B relies on the English alphabet and may not be suitable for reading-disabled, illiterate, elderly and dyslexic participants or non-native speakers whose first language uses a different writing system (Guo, 2022; Simfukwe et al., 2022; Waggestad et al., 2023); sensitive to practice effects with 1-week intervals (Buck et al., 2008); the test measures multiple executive functions, resulting in low specificity (Linari et al., 2022)	with PD (Houvenaghel et al., 2018 — with STN-DBS; Barbosa et al., 2017); adults with ALS (Kasper et al., 2015); a mixed sample of neurological / psychiatric patients and healthy adults (Whiteside et al., 2016)		TMT across populations implies its sensitivity to distributed prefrontal network integrity rather than a single shared mechanism. TMT-B's dependence on the Roman alphabet creates a structural mismatch with non-alphabetic or low-literacy populations, possibly resulting in null findings in some bilingual and cross-cultural samples. Practice effects within serial assessments could suppress correlations in longitudinal fluency research. For future research, alphabet literacy may be included as a covariate in cross-cultural designs.
**Wisconsin Card Sorting Test** (WCST: Grant and Berg, 1948)<br><br>switching; working memory	May trigger an emotional response (Howieson, 2019); less sensitive to executive differences in healthy young adults; practice effects may undermine construct validity; different	healthy adults (Galtier et al., 2017); stroke survivors (Turunen et al., 2016); adults with PD (Galtier et al., 2017); adults with RRMS (Svindt et al., 2023); a mixed sample of neurological / psychiatric patients and healthy adults	healthy adults (Turunen et al., 2016; Gabrić and Vandek, 2022; Svindt et al., 2023); adults with PD (Houvenaghel et al., 2018 — with STN-DBS); healthy children and adolescents (Wen, 2019)	Cognitive flexibility — updating response rules based on feedback — underlies both WCST (shifting sorting criteria) and PF (abandoning exhausted subcategories and adopting new search strategies). The pattern of correlations primarily in clinical groups suggests that in unimpaired individuals, set-shifting during fluency relies on

	underlying factor structure may be inherent in different populations (Suchy et al., 2017)	(Whiteside et al., 2016)		automated retrieval routines that bypass effortful flexibility. Practice effects and differential factor structure across populations further complicate interpretation. WCST seems to be better for detecting flexibility deficits in clinical groups rather than explaining fluency variance in healthy participants.
**A composite score** derived from multiple tests, at least one of which belonging to the core seven listed above<br><br>inhibition, shifting, updating		healthy adults and adolescents (Gustavson et al., 2019)		Composite executive scores reduce construct-specific noise providing a more stable estimate of shared variance with PVF. The single eligible study found a robust correlation, consistent with a general executive factor contributing to fluency across development. In future research, component-level analysis may be added to disentangle the construct-specific from some shared executive variance.

Part 2. Language

-

Limitations
A single test paradigm may be linked to a range of constructs with varying complexity (sometimes several at a time) in different studies; the actual construct validity of each paradigm may not be accounted for in the study (Morkovina et al., 2023; Morkovina et al., 2024); the complexity of each construct is not addressed from the linguistic point of view (e.g. ‘vocabulary knowledge’: see Milton and Fitzpatrick, 2014); a single construct may be targeted via different paradigms without accounting for its linguistic underpinnings.

Paradigm / Examples	Presumed abilities (according to the articles listed)	Correlations present	Correlations absent or not significant	Phonemic Fluency Implications
**Confrontation naming** (BNT, GNT, BS (German))	semantic retrieval; lexical access; word-finding; naming ability	healthy adults (González-Burgos et al., 2019 — older and with high cognitive reserve; González-Burgos et al., 2021); Carpenter et al., 2020 — bilinguals tested in L1 and L2 fluency, naming in L1; tested in L2 fluency: naming in L2); adults with MS (Delgado-Álvarez et al., 2021); adults with aphasia (Rossetti et al., 2019 — single case, with TMS; Carpenter et al., 2020 — the same as in healthy bilinguals); a mixed sample of neurological / psychiatric patients and healthy adults (Whiteside et al., 2016)	healthy adults (Shao et al., 2014; Stolwyk et al., 2015; Kasper et al., 2015; González-Burgos et al., 2019 — younger adults, ones with low cognitive reserve; Carpenter et al., 2020 — bilinguals tested in L1 fluency, naming in L2); adults with ALS (Kasper et al., 2015); glioma patients (Collée et al., 2023); adults with aphasia (Carpenter et al., 2020 — the same as in healthy bilinguals)	Both confrontation naming and phonemic fluency engage phonological encoding and lexical access, but differ in retrieval mode: naming is cue-driven (visual stimulus activates a target lexeme), while phonemic fluency is internally driven (a phonological cue initiates a self-generated search). Correlations could be explained by the lexical bottleneck in retrieval, as seen in older adults with high cognitive reserve and in clinical populations with degraded lexical networks. Absence of correlation in younger healthy adults and some aphasia subgroups may reflect route-specific dissociation: cue-driven and internally initiated retrieval recruit partially distinct pathways.
**Word definition** (WAIS-R, WAIS-III,	vocabulary; word knowledge and language	healthy adults (Tombaugh et al., 1999; Aita et al., 2019); adults with MS (Lebkuecher		Vocabulary depth tests tap crystallised lexical-semantic knowledge (the mental lexicon). Phonemic fluency production is facilitated by a dense,

WAIS-IV, WASI)	ability: crystalized intelligence, language development, and general knowledge of word meanings	et al., 2021); a mixed sample of neurological / psychiatric patients and healthy adults (Whiteside et al., 2016)		well-organised semantic network that enables rapid traversal along the phonologically linked lexical nodes. The near-universal correlation of word definition scores with fluency across populations reflects this stable relationship: broader vocabulary supports higher fluency output largely independently of any executive control fluctuations. Vocabulary tests are less susceptible to speed or inhibitory confounds than most executive measures, making them useful covariates when researchers seek to isolate the executive contribution to fluency after controlling for baseline lexical knowledge.
**Receptive vocabulary test** (BPVS-III, PPVT-4, PMA Vocabulary)	vocabulary knowledge	healthy adults (Filippi et al., 2022 — monolingual and multilingual); healthy children (Filippi et al., 2022 — monolingual and multilingual; Hoang et al., 2021); healthy adolescents (Gustavson et al., 2019)	healthy adults (Shao et al., 2014)	Receptive vocabulary measures the breadth of the passive lexicon — the range of words a person can recognise. Correlations with phonemic fluency in children and multilingual adults might reflect that a larger accessible lexical store increases the pool from which a phonemic cue can trigger matches. Absent correlations in some adult studies suggest that once a sufficient passive lexical threshold is reached, production fluency is no longer vocabulary-limited but becomes constrained by access speed, phonological retrieval efficiency, or inhibitory control. The receptive-expressive vocabulary gap is especially relevant in multilingual and L2 speakers, where passive knowledge does not guarantee productive access.
**Single word oral reading** (NART, NART-R)	verbal intelligence	healthy adults (Stolwyk et al., 2015)		NART estimates premorbid verbal intelligence via recognition and pronunciation of irregular words — a skill heavily dependent on prior educational and linguistic exposure. Its correlation with

				phonemic fluency most plausibly reflects a shared variance in general verbal ability and educational attainment rather than a specific link to phonological retrieval or executive control. NART is most useful as a covariate to estimate premorbid ability in clinical populations, thereby controlling for the baseline verbal competence that systematically predicts fluency output.
**Other** (AAT Repetition, Token Test)	AAT Repetition — Phonology; TT — Language comprehension	glioma patients (Collée et al., 2023 — Token Test)	glioma patients (Collée et al., 2023 — AAT Repetition)	The Token Test correlates with phonemic fluency in glioma patients, suggesting that integrity of the perisylvian language network broadly supports both receptive processing and expressive phonemic search — a potential reflection of common neural substrate rather than a direct cognitive link. The lack of correlation for AAT Repetition (phonological encoding) may indicate that phonological encoding per se is dissociable from the phonological retrieval demands of fluency, a clinically important distinction. The Token Test correlation may be connected with tumour location rather than a shared cognitive mechanism, limiting generalisability.
**A composite score** derived from multiple tests (e.g. WASI-V + AFQT-V) or a test battery (e.g. BDAE, PALPA, BAT)	depending on the chosen set of tests: lexical-semantic processing; vocabulary; general semantic knowledge and expressive language	healthy adults Carpenter et al., 2020 — bilinguals tested in L1 and L2 fluency, several PALPA tests in L1; tested in L1 fluency, several PALPA tests in L2; tested in L1 and L2 fluency, several BAT tests in L1 and L2); adults with aphasia (Vuković and	healthy adults (Gustavson et al., 2019; Carpenter et al., 2020 — bilinguals tested in L2 fluency, several PALPA tests in L2); adults with aphasia (Carpenter et al., 2020 — the same as in healthy bilinguals; adults with aphasia (Vuković and Chen, 2024 — Broca's aphasia);	Composite language scores reduce paradigm-specific noise and measurement error, potentially revealing those fluency correlations that individual tests obscure. The complex bilingual-aphasia findings illustrate that the relationship between composite language ability and fluency is profoundly modulated by testing language (L1 vs. L2), proficiency level, and aphasia type — not reducible to a single correlation coefficient. PALPA and BAT batteries are supposed to tap multiple

		Chen, 2024 — transcortical motor aphasia; Carpenter et al. 2020 — the same as in healthy bilinguals); a mixed sample of children with SLI and healthy children (Henry et al., 2015)		processing levels (phonological, lexical-semantic, syntactic), and their overlap with fluency depends on subtest selection. Composite language scores are most informative when factor structure is reported, L1 and L2 performance is analysed separately for bilingual participants, and subtest selection is justified against the specific fluency construct under investigation.

Supplementary Table References

1. Aita, S. L., Beach, J. D., Taylor, S. E., Borgogna, N. C., Harrell, M. N., Hill, B. D. (2019). Executive, language, or both? An examination of the construct validity of verbal fluency measures. *Appl. Neuropsychol. Adult* 26(5), 441–451. doi: 10.1080/23279095.2018.1439830
2. D'Antuono, G., La Torre, F. R., Marin, D., Antonucci, G., Piccardi, L., Guariglia, C. (2017). Role of working memory, inhibition, and fluid intelligence in the performance of the Tower of London task. *Appl. Neuropsychol. Adult* 24(6), 548–558. doi: 10.1080/23279095.2016.1225071
3. Ardila, A., Ostrosky, F. (2022). What do neuropsychological tests assess? *Applied Neuropsychology: Adult* 29(1), 1–9. doi: 10.1080/23279095.2019.1699099
4. Army Individual Test Battery. (1944). *Manual of Directions and Scoring*. Washington, DC: War Department, Adjutant General's Office.
5. Baggetta, P., Alexander, P. A. (2016). Conceptualization and operationalization of executive function. *Mind Brain Educ.* 10(1), 10–33. doi: 10.1111/mbe.12100.
6. Barbosa, A. F., Voos, M. C., Chen, J., Francato, D. C. V., Souza, C. D. O., Barbosa, E. R., Chien, H.F., Mansur, L. L. (2017). Cognitive or Cognitive-Motor Executive Function Tasks? Evaluating Verbal Fluency Measures in People with Parkinson's Disease. *Biomed Res. Int.* 2017(1), 7893975. doi: 10.1155/2017/7893975
7. Buck, K. K., Atkinson, T. M., Ryan, J. P. (2008). Evidence of practice effects in variants of the Trail Making Test during serial assessment. *J. Clin. Exp. Neuropsychol.* 30(3), 312–318. doi: 10.1080/13803390701390483
8. Carpenter, E., Rao, L., Peñaloza, C., Kiran, S. (2020). Verbal fluency as a measure of lexical access and cognitive control in bilingual persons with aphasia. *Aphasiology* 34(11), 1341–62. doi: 10.1080/02687038.2020.1759774
9. Chan, E., MacPherson, S. E., Robinson, G., Turner, M., Lecce, F., Shallice, T., Cipolotti, L. (2015). Limitations of the trail making test part-B in assessing frontal executive dysfunction. *J. Int. Neuropsychol. Soc.* 21(2), 169–174. doi: 10.1017/S135561771500003X
10. Collée, E., Berg, E., Visch-Brink, E., Vincent, A., Dirven, C., Satoer, D. (2023). Differential contribution of language and executive functioning to verbal fluency performance in glioma patients. *J. Neuropsychol.* 18, 19–40. doi: 10.1111/jnp.12356
11. Criaud, M., Boulinguez, P. (2013). Have we been asking the right questions when assessing response inhibition in go/no-go tasks with fMRI? A meta-analysis and critical review. *Neurosci. Biobehav. Rev.* 37(1), 11–23. doi: 10.1016/j.neubiorev.2012.11.003
12. Delgado-Álvarez, A., Matias-Guiu, J. A., Delgado-Alonso, C., Hernández-Lorenzo, L., Cortés-Martínez, A., Vidorreta, L., Montero-Escribano, P., Pytel, V., Matias-Guiu, J. (2021).

Cognitive Processes Underlying Verbal Fluency in Multiple Sclerosis. *Front. Neurol.* 11, 629183. doi: 10.3389/fneur.2020.629183
13. Diamond, A. (2013). Executive functions. *Ann. Rev. Psychol.* 64(1), 135–168. doi: 10.1146/annurev-psych-113011-143750
14. Donders, F. C. (1868). Over de snelheid van psychische processen. *Onderzoekingen gedaan in het Physiologisch Laboratorium der Utrechtsche Hoogeschool (1968–1869)* 2, 92–120.
15. Donders, F. C. (1969). On the speed of mental processes. *Acta Psychol.* 30, 412–431. doi: 10.1016/0001-6918(69)90065-1
16. Duan, C., Chong, Y., Gong, J., Wu, Q., Sun, J., Zheng, C., Li, Z., Xia, L., Cheng, Z., Zhang, P., Xia, W. (2025). An fNIRS-based investigation of cerebral hemodynamic responses during verbal fluency task and n-back task in individuals with mild cognitive impairment. *Front. Neurol.* 16, 1571964. doi: 10.3389/fneur.2025.1571964
17. Eagle, D.M., Bari, A., Robbins, T.W. (2008). The neuropsychopharmacology of action inhibition: cross-species translation of the stop-signal and go/no-go tasks. *Psychopharmacology* 199(3), 439–456. doi: 10.1007/s00213-008-1127-6
18. Egeland, J., Lund, O., Raudeberg, R. (2026). Measuring working memory span with WAIS-IV: Digit sequence is the superior span test. *Applied Neuropsychology: Adult*, 33(1), 65–72. doi: 10.1080/23279095.2024.2330998
19. Faria, C. D. A., Alves, H. V. D., Charchat-Fichman, H. (2015). The most frequently used tests for assessing executive functions in aging. *Dement. Neuropsychol.* 9(2), 149–155. doi: 10.1590/1980-57642015DN92000009
20. Filippi, R., Ceccolini, A., Bright, P. (2022). Trajectories of verbal fluency and executive functions in multilingual and monolingual children and adults: A cross-sectional study. *Q. J. Exp. Psychol.* 75(1), 130–147. doi: 10.1177/17470218211026792
21. Gabrić, P., Vandek, M. (2022). Performance on verbal fluency tasks depends on the given category/letter: Preliminary data from a multivariable analysis. *medRxiv*, 1–37. doi: 10.1101/2021.12.30.21268567
22. Gajewski, P.D., Hanisch, E., Falkenstein, M., Thönes, S., Wascher, E. (2018). What Does the n-Back Task Measure as We Get Older? Relations Between Working-Memory Measures and Other Cognitive Functions Across the Lifespan. *Front. Psychol.* 9, 2208. doi: 10.3389/fpsyg.2018.02208
23. Galtier, I., Nieto, A., Lorenzo, J. N., Barroso, J. (2017). Mild cognitive impairment in Parkinson's disease: clustering and switching analyses in verbal fluency test. *J. Int. Neuropsychol. Soc.* 23(6), 511–520. doi: 10.1017/S1355617717000297
24. Galton, F. (1887). Supplementary notes on 'prehension' in idiots. *Mind* 12, 79–82.
25. De Giacomo, A., Murri, A., Matera, E., Pompamea, F., Craig, F., Giagnotti, F., Bartoli, R., Quaranta, N. (2021). Executive functions and deafness: Results in a group of cochlear implanted children. *Audiol. Res.* 11(4), 706–717. doi: 10.3390/audiolres11040063
26. Giovannoli, J., Martella, D., Federico, F., Pirchio, S., Casagrande, M. (2020). The Impact of Bilingualism on Executive Functions in Children and Adolescents: A Systematic Review Based on the PRISMA Method. *Front. Psychol.* 11, 574789. doi: 10.3389/fpsyg.2020.574789
27. González-Burgos, L., Hernández-Cabrera, J. A., Westman, E., Barroso, J., Ferreira, D. (2019). Cognitive compensatory mechanisms in normal aging: A study on verbal fluency and the contribution of other cognitive functions. *Aging* 11(12), 4090–4106. doi: 10.18632/aging.102040
28. González-Burgos, L., Pereira, J. B., Mohanty, R., Barroso, J., Westman, E., Ferreira. D. (2021). Cortical networks underpinning compensation of verbal fluency in normal aging. *Cereb, Cortex* 31(8), 3832–45. doi: 10.1093/cercor/bhab052
29. Grant, D. A., Berg, E. (1948). A behavioral analysis of degree of reinforcement and ease of shifting to new responses in a Weigl-type card-sorting problem. *J. Exp. Psychol.* 38(4), 404–411. doi: 10.1037/h0059831

30. Guo, Y. (2022). A selective review of the ability for variants of the Trail Making Test to assess cognitive impairment. *Appl. Neuropsychol. Adult* 29(6), 1634–1645. doi: 10.1080/23279095.2021
31. Gustavson, D. E., Panizzon, M. S., Franz, C. E., Reynolds, C. A., Corley, R. P., Hewitt, J. K., Lyons, M. J., Kremen, W. S., Friedman, N. P. (2019). Integrating verbal fluency with executive functions: Evidence from twin studies in adolescence and middle age. *J. Exp. Psychol. Gen.* 148(12), 2104–2119. doi: 10.1037/xge0000589
32. Henry, L., Messer, D. J., Nash, G. (2015). Executive functioning and verbal fluency in children with language difficulties. *Learn. Instr.* 39, 137–147. doi: 10.1016/j.learninstruc.2015.06.001
33. Hoang, G. T. H., Baten, K., De Cuypere, L., Hoang, T. T., Taverniers, M. (2021). An exploratory study of predictors of vocabulary knowledge of Vietnames preschool-age children in a city: Predictors of vocabulary knowledge in Vietnamese preschoolers. *Dutch J. Appl. Linguist*. 10, 1–24. doi: 10.51751/dujal9538
34. Houvenaghel, J. F., Drapier, S., Duprez, J., Robert, G. H., Riou, A., Drapier, D., Sauleau, P., Vérin, M. (2018). Effects of continuous subcutaneous apomorphine infusion in Parkinson's disease without cognitive impairment on motor, cognitive, psychiatric symptoms and quality of life. *J. Neurol. Sci.* 395, 113–118. doi: 10.1016/j.jns.2018.10.010
35. Howieson, D. (2019). Current limitations of neuropsychological tests and assessment procedures. *Clin. Neuropsychol.* 33(2), 200–208. doi: 10.1080/13854046.2018.1552762
36. Jacobs, J. (1887). Experiments on “prehension”. *Mind* 12, 75–79.
37. Jaeggi, S. M., Buschkuehl, M., Perrig, W. J., Meier, B. (2010). The concurrent validity of the N-back task as a working memory measure. *Memory* 18(4), 394–412. doi: 10.1080/09658211003702171
38. Kane, M. J., Conway, A. R., Miura, T. K., Colflesh, G. J. (2007). Working memory, attention control, and the N-back task: a question of construct validity. *J. Exp. Psychol. Learn. Mem. Cogn.* 33(3), 615–622. doi: 10.1037/0278-7393.33.3.615
39. Kasper, E., Schuster, C., Machts, J., Bittner, D., Vielhaber, S., Benecke, R., Teipel, S., Prudlo, J. (2015). Dysexecutive functioning in ALS patients and its clinical implications. *Amyotroph. Lateral Scler. Frontotemporal Degener.* 16(3–4), 160–171. doi: 10.3109/21678421.2015.1026267
40. Kavé, G., Sapir-Yogev, S. (2020). Associations between memory and verbal fluency tasks. *J. Commun. Disord.* 83, 105968. doi: 10.1016/j.jcomdis.2019.105968
41. Kirchner, W. K. (1958). Age differences in short-term retention of rapidly changing information. *J. Exp. Psychol*. 55(4), 352–358. doi: 10.1037/h0043688
42. Koppenol-Gonzalez, G. V., Bouwmeester, S., Boonstra, A. M. (2010). Understanding planning ability measured by the Tower of London: An evaluation of its internal structure by latent variable modeling. *Psychol. Assess.* 22(4), 923–934. doi: 10.1037/a0020826
43. Kraan, C., Stolwyk, R. J., Testa, R. (2013). The abilities associated with verbal fluency performance in a young, healthy population are multifactorial and differ across fluency variants. *Appl. Neuropsychol. Adult* 20(3), 159–168. doi: 10.1080/09084282.2012.670157
44. Lebkuecher, A. L., Chiaravalloti, N. D., Strober, L. B. (2021). The role of language ability in verbal fluency of individuals with multiple sclerosis. *Mult. Scler. Relat. Disord.* 50, 102846. doi: 10.1016/j.msard.2021.102846
45. Linari, I., Juantorena, G. E., Ibáñez, A., Petroni, A., Kamienkowski, J. E. (2022). Unveiling Trail Making Test: visual and manual trajectories indexing multiple executive processes. *Sci. Rep.* 12(1), 14265. doi: 10.1038/s41598-022-16431-9
46. Mackworth, J. F. (1959). Paced memorizing in a continuous task. *J. Exp. Psychol*. 58, 206–211. doi: 10.1037/h0049090
47. Milton, J., Fitzpatrick, T. (2014). *Dimensions of vocabulary knowledge*. Basingstoke: Palgrave Macmillan.

48. Morkovina, O. I., Gishkaeva, L. N., Sharapkova, A. A. (2023). Picture naming test: linguistic challenges of the method and ways to solve them. *Russ. J. Linguist.* 27(3), 715–744. doi: 10.22363/2687-0088-34934
49. Morkovina, O., Manukyan, P., Sharapkova, A. (2024). Picture naming test through the prism of cognitive neuroscience and linguistics: adapting the test for cerebellar tumor survivors—or pouring new wine in old sacks? *Front. Psychol.* 15, 1332391. doi: 10.3389/fpsyg.2024.1332391
50. Patra, A., Bose, A., Marinis, T. (2020a). Lexical and Cognitive Underpinnings of Verbal Fluency: Evidence from Bengali-English Bilingual Aphasia. *Behav. Sci.* 10(10), 155. doi: 10.3390/bs1010
51. Patra, A., Bose, A., Marinis, T. (2020b). Performance difference in verbal fluency in bilingual and monolingual speakers. *Biling. Lang. Cogn.* 23(1), 204–218. doi: 10.1017/S1366728918001098
52. Rossetti, A., Malfitano, C., Malloggi, C., Banco, E., Rota, V., Tesio, L. (2019). Phonemic fluency improved after inhibitory transcranial magnetic stimulation in a case of chronic aphasia. *Int. J. Rehab. Res*. 42(1), 92–95. doi: 10.1097/MRR.0000000000000322
53. Schmiedek, F., Lövdén, M., Lindenberger, U. (2014). A task is a task is a task: putting complex span, *n*-back, and other working memory indicators in psychometric context. *Front. Psychol.* 5, 1475. doi: 10.3389/fpsyg.2014.01475
54. Shallice, T. (1982). Specific impairments of planning. *Philos. Trans. R. Soc. Lond. B Biol. Sci.* 298(1089), 199–209. doi: 10.1098/rstb.1982.0082
55. Shao, Z., Roelofs, A., Meyer, A. S. (2014). Predicting naming latencies for action pictures: Dutch norms. *Behav. Res. Methods* 46(1), 274–283. doi: 10.3758/s13428-013-0358-6
56. Simfukwe, C., Youn, Y. C., Kim, S. Y., An, S. S. (2022). Digital trail making test-black and white: Normal vs MCI. *Appl. Neuropsychol. Adult* 29(6), 1296–1303. doi: 10.1080/23279095.2021.1871615
57. Simmonds, D. J., Pekar, J. J., Mostofsky, S. H. (2008). Meta-analysis of Go/No-go tasks demonstrating that fMRI activation associated with response inhibition is task-dependent. *Neuropsychologia* 46(1), 224–232. doi: 10.1016/j.neuropsychologia.2007.07.015
58. Stavroussi, P., Andreou, G., Karagiannopoulou, D. (2016). Verbal fluency and verbal short-term memory in adults with Down syndrome and unspecified intellectual disability. *Int. J. Disab. Dev. Educ.* 63(1), 122–139. doi: 10.1080/1034912X.2015.1111307
59. Stolwyk, R., Bannirchelvam, B., Kraan, C., Simpson, K. (2015). The cognitive abilities associated with verbal fluency task performance differ across fluency variants and age groups in healthy young and old adults. *J. Clin. Exp. Neuropsychol.* 37(1), 70–83. doi: 10.1080/13803395.2014.988125
60. Stroop, J. R. (1935). Studies of interference in serial verbal reactions. *J. Exp. Psychol.* 18(6), 643–662. doi: 10.1037/h0054651
61. Suchy, Y., Niermeyer, M. A., Ziemnik, R. E. (2017). "Assessment of executive functions in research," in *Executive functions in health and disease*, ed. E. Goldberg (Academic Press), 197–216.
62. Svindt, V., Gosztolya, G., Gráczi, T. E. (2023). Narrative recall in relapsing-remitting multiple sclerosis: A potentially useful speech task for detecting subtle cognitive changes. *Clin. Linguist. Phon.* 37(4–6), 549–566. doi: 10.1080/02699206.2023.2170830
63. Tamez, E., Myerson, J., Morris, L., White, D. A., Baum, C., Connor, L. T. (2011). Assessing executive abilities following acute stroke with the trail making test and digit span. *Behav. Neurol.* 24(3), 177–185. doi: 10.3233/BEN-2011-0328
64. Tombaugh, T. N., Kozak, J., Rees, L. (1999). Normative data stratified by age and education for two measures of verbal fluency: FAS and animal naming. *Arch. Clin. Neuropsychol.* 14(2), 167–177.
65. Treviño, M., Zhu, X., Lu, Y.Y., Scheuer, L.S., Passell, E., Huang, G.C., Germine, L.T., Horowitz, T.S. (2021). How do we measure attention? Using factor analysis to establish

construct validity of neuropsychological tests. *Cogn. Res. Princ. Implic.* 6, 51. doi: 10.1186/s41235-021-00313-1
66. Turunen, K. E., Laari, S. P., Kauranen, T. V., Mustanoja, S., Tatlisumak, T., Poutiainen, E. (2016). Executive impairment is associated with impaired memory performance in working-aged stroke patients. *J. Int. Neuropsychol. Soc.* 22(5), 551–560. doi: 10.1017/S1355617716000205
67. Tyburski, E., Kerestey, M., Kerestey, P., Radoń, S., Mueller, S. T. (2021). Assessment of motor planning and inhibition performance in non-clinical sample—reliability and factor structure of the Tower of London and Go/No Go computerized tasks. *Brain Sci.* 11(11), 1420. doi: 10.3390/brainsci11111420
68. Ucheagwu, V., Odilora, C., Ugokwe-Joseph, R., Giordani, B. (2024). Factor Structure and Internal Consistency of the National Alzheimer Coordinating Center's Uniform Data Set Version 3 Neuropsychological Test Battery (UDSNB 3.0): The Nigeria Sample. *Alzheimer Dis. Assoc. Disord.* 38(3), 265–270. doi: 10.1097/WAD.0000000000000630
69. Unterrainer, J. M., Rahm, B., Kaller, C. P., Leonhart, R., Quiske, K., Hoppe-Seyler, K., Meier, C., Müller, C., Halsband, U. (2004). Planning abilities and the Tower of London: is this task measuring a discrete cognitive function? *J. Clin. Exp. Neuropsychol.* 26(6), 846–856. doi: 10.1080/13803390490509574
70. Unterrainer, J. M., Rahm, B., Leonhart, R., Ruff, C. C., Halsband, U. (2003). The Tower of London: The impact of instructions, cueing, and learning on planning abilities. *Cogn. Brain Res.* 17(3), 675–683. doi: 10.1016/s0926-6410(03)00191-5
71. Villalobos, D., Torres-Simón, L., Pacios, J., Paúl, N., Del Río, D. (2023). A Systematic Review of Normative Data for Verbal Fluency Test in Different Languages. *Neuropsychol. Rev.* 33(4). doi: 10.1007/s11065-022-09549-0
72. Vuković, M., Chen, L. (2024). Language and executive functions in patients with transcortical motor aphasia and Broca's aphasia. *Clin. Linguist. Phon.* 39(6–8), 765–783. doi: 10.1080/02699206.2024.2393
73. Waggestad, T. H., Kirsebom, B. E., Strobel, C., Wallin, A., Eckerström, M., Fladby, T., Egeland, J. (2023). Improving validity of the trail making test with alphabet support. *Front. Psychol.* 14, 1227578. doi: 10.3389/fpsyg.2023.1227578
74. Wen, Y. (2019). *Exploring the structure and the roles of executive functions in typically developing children and children with autism spectrum disorder* [Doctoral dissertation] Newcastle: Newcastle University.
75. Whiteside, D. M., Kealey, T., Semla, M., Luu, H., Rice, L., Basso, M. R., Roper, B. (2016). Verbal fluency: Language or executive function measure? *Appl. Neuropsychol. Adult* 23(1), 29–34. doi: 10.1080/23279095.2015.1004574
76. Wright, L., Lipszyc, J., Dupuis, A., Thayapararajah, S. W., Schachar, R. (2014). Response inhibition and psychopathology: A meta-analysis of go/no-go task performance. *J. Abnorm. Psychol.* 123(2), 429–439. doi: 10.1037/a0036295
77. Zook, N. A., Davalos, D. B., DeLosh, E. L., Davis, H. P. (2004). Working memory, inhibition, and fluid intelligence as predictors of performance on Tower of Hanoi and London tasks. *Brain Cogn.* 56(3), 286–292. doi: 10.1016/j.bandc.2004.07.003
